# Advances in the pharmacological effects and molecular mechanisms of emodin in the treatment of metabolic diseases

**DOI:** 10.3389/fphar.2023.1240820

**Published:** 2023-10-31

**Authors:** Linyuan Yu, Yongliang Zhao, Yongli Zhao

**Affiliations:** ^1^ Department of Traditional Chinese Medicine, Chengdu Integrated TCM and Western Medicine Hospital, Chengdu, China; ^2^ Department of Pharmacy, Sichuan Second Hospital of TCM, Chengdu, China; ^3^ Nursing Department, Affiliated Hospital of Zunyi Medical University, Zunyi, China

**Keywords:** emodin, metabolic diseases, pharmacological effects, glycolipid metabolism, molecular mechanisms, toxicological

## Abstract

*Rhubarb palmatum* L.*, Polygonum multijiorum* Thunb*.,* and *Polygonum cuspidatum* Sieb. Et Zucc. are traditional Chinese medicines that have been used for thousands of years. They are formulated into various preparations and are widely used. Emodin is a traditional Chinese medicine monomer and the main active ingredient in *Rhubarb palmatum* L.*, Polygonum multijiorum* Thunb*.,* and *Polygonum cuspidatum* Sieb. Et Zucc. Modern research shows that it has a variety of pharmacological effects, including promoting lipid and glucose metabolism, osteogenesis, and anti-inflammatory and anti-autophagy effects. Research on the toxicity and pharmacokinetics of emodin can promote its clinical application. This review aims to provide a basis for further development and clinical research of emodin in the treatment of metabolic diseases. We performed a comprehensive summary of the pharmacology and molecular mechanisms of emodin in treating metabolic diseases by searching databases such as Web of Science, PubMed, ScienceDirect, and CNKI up to 2023. In addition, this review also analyzes the toxicity and pharmacokinetics of emodin. The results show that emodin mainly regulates AMPK, PPAR, and inflammation-related signaling pathways, and has a good therapeutic effect on obesity, hyperlipidemia, non-alcoholic fatty liver disease, diabetes and its complications, and osteoporosis. In addition, controlling toxic factors and improving bioavailability are of great significance for its clinical application.

## Graphical Abstract

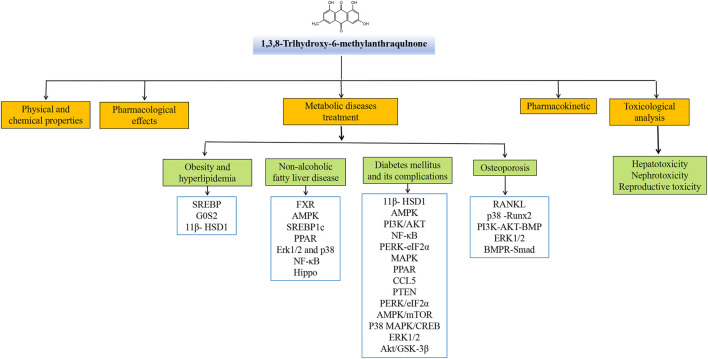

## 1 Introduction

Emodin belongs to the anthraquinone compound and is the main indicator component of *Rhubarb palmatum* L. (RP)*, Polygonum multijiorum* Thunb*.* (PM), and *Polygonum cuspidatum* Sieb. Et Zucc. (PC) of the Polygonaceae family. RP*,* PM, and PC are included in the “Chinese Pharmacopoeia 2020,” but their functions are different. RP mainly has the effect of heat-clearing and diuresis-promoting, PM is mainly used to tonify the liver and kidneys, and PC mainly has the effect of removing dampness and reducing jaundice (Lin et al., 2015; Cao et al., 2017; Xu H. et al., 2021). RP*,* PM, and PC are often made into preparations in China, such as Jiangzhining capsules, Danning tablets, Niuhuang Jiedu tablets, and Reyaning granules. These preparations are sold in the Chinese market. Therefore, emodin, as the main pharmacological component of RP*,* PM, and PC has been widely studied. Modern research has found that emodin has pharmacological effects such as being anticancer (Hsu and Chung, 2012), anti-inflammatory, and analgesic (Yang et al., 2023), as well as providing protection to the kidneys and having lipid-lowering properties (He L. F. et al., 2022; Liu Y. et al., 2022).

In our previous study, we identified emodin as an active substance with the ability to lower blood lipids and regulate glucose utilization (Yu L. et al., 2020). Furthermore, emodin is a regulator of AMP-activated protein kinase (AMPK) and peroxisome proliferator-activated receptors (PPARs) (Chen et al., 2012; Li W. et al., 2021). AMPK and PPARs are the main targets involved in metabolic diseases, such as obesity, non-alcoholic fatty liver disease, atherosclerosis, and diabetes (Fruchart et al., 2001; Day et al., 2017). AMPK has been shown to stimulate the breakdown of macromolecules for energy production by increasing glucose utilization and mobilizing lipid stores and the turnover of macromolecular pathways through autophagy (Herzig and Shaw, 2018). PPARγ improves insulin sensitivity and glucose metabolism by affecting lipid metabolism (Montaigne et al., 2021). In addition, emodin also has a good anti-inflammatory effect, so it has a good therapeutic effect on lipid and glucose metabolism disorders and subsequent inflammatory diseases. Therefore, it is speculated that emodin may participate in metabolic diseases through its regulatory effects on energy and inflammation. However, there is a lack of a comprehensive summary of emodin in the treatment of metabolic diseases, and the toxicity of emodin is well known, which hinders its further development. Therefore, this review provides a systematic review of the treatment of metabolic diseases with emodin and the signal pathways involved, and analyzes its common toxicities and pharmacokinetics. We searched the Web of Science, PubMed and CNKI databases up to August 2023, with the aim of providing guidance and evidence for emodin research on metabolic diseases.

## 2 Physical and chemical properties of emodin

**FIGURE 1:**
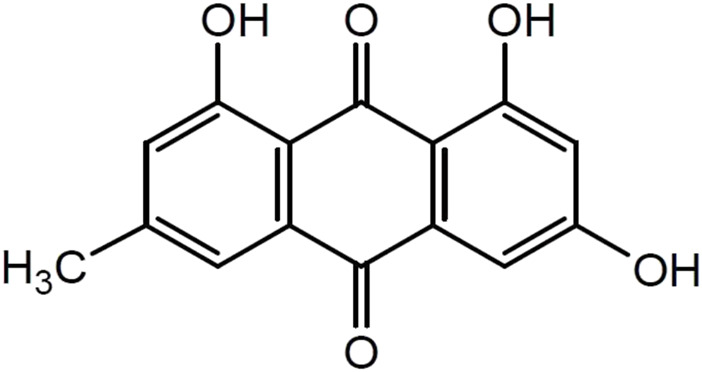
Chemical Structure of emodin.

Emodin (1,3,8-trihydroxy-6-methylanthraquinone) is a natural anthraquinone compound widely found in RP*,* PM, PC, and other plants and Chinese herbal medicines, and it is used as the main quality detection index in the 2020 edition of the Chinese Pharmacopoeia. Emodin is an orange-yellow long needle-shaped crystal with a molecular weight of 270.24 and a density of 1.328 g/cm^3^. The chemical structure of emodin is shown in Figure 1. The physical and chemical properties of emodin are shown in Table 1 (Data comes from www.chemicalbook.com/).

**TABLE 1:** Physical and chemical properties of emodin.

Name	1,3,8-Trihydroxy-6-methylanthraquinone
Alias	6-Methyl-1,3,8-trihydroxyanthraquinone
Source	*Rhubarb palmatum* L., Polygonum multiflorum Thunb*.,* and *Polygonum cuspidatum* Sieb. Et Zucc
CAS number	518-82-1
EINECS number	208-258-8
Compound type	anthraquinone
Molecular formula	C15H10O5
Molecular weight	270.24
Properties	powder
Color	orange
Solubility	<0.1 g/100 mL at 19°C
Density	1.3280 g/cm3
pKa	6.39 ± 0.20(Predicted)
Boiling point	373.35°C at 760 mmHg
Flash point	322.8°C ± 23.6°C
Vapor pressure	0.0 ± 1.7 mmHg at 25°C
Refractivity	1.745
Polar surface area	94.83
LogP	3.641 (est)
Storage conditions	2°C–8°C, cool, dry, and sealed

## 3 Pharmacological properties

Emodin has a variety of pharmacological effects, including anti-inflammatory, anti-tumor, antibacterial, immune enhancement, lipid-lowering, blood glucose-lowering, kidney protection, etc. Emodin can regulate signal transduction pathways with various pharmacological properties. For example, anti-inflammatory activity mainly involves NLRP3, NF-κB, mTOR, Notch, and JAK1/STAT3; anti-tumor activity mainly involves PI3K/AKT, NF-κB, TRAF6, and EMT; antibacterial activity mainly involves NF-κB and PPARγ; and their pharmacological effects and mechanisms are shown in Table 2. The immune-enhancing effect of emodin is mainly related to regulating intestinal function and repairing the intestinal barrier (Zhou et al., 2021; Mabwi et al., 2023). This article mainly reviews the pharmacological effects and mechanisms of emodin related to anti-metabolic diseases, including lowering lipids, lowering blood glucose, and protecting kidneys, as shown in Table 3.

**TABLE 2:** Pharmacological effects and mechanisms of emodin.

Pharmacological effects	Models	Dosing method	Dosage of administration	Molecular mechanisms	References
Anti-inflammatory	Rat model of ischemia-reperfusion injury	i.p	40 mg/kg	Inactivating the TLR4/MyD88/NF-κB/NLRP3 pathway	Jin et al. (2023)
	Human nucleus pulposus cells (NPCs) were stimulated with IL-1β to induce inflammation	indicated	20 μM	Inhibiting ROS-mediated NF-κB activation	Zhu et al. (2023)
	Establishment of experimental autoimmune encephalomyelitis rat model through immune induction	i.p	20 mg/kg	Regulating the SIRT1/PGC-1α/NLRP3 signaling pathway and inhibiting microglial inflammation	Cui et al. (2023)
	Mouse model of myocardial ischemia/reperfusion injury	oral	10 mg/kg	Modulating the RUNX1/miR-142-3p/DRD2 pathway inhibits NF-κB-triggered inflammation	Zhang et al. (2022c)
	Type II collagen-induced arthritis model in mice	i.p	10 mg/kg	Activating AMPK to inhibit the inflammatory response of NLRP3	Cheng et al. (2022)
	Acute pancreatitis (AP) rat model induced by taurocholate	oral	10 mg/kg	Suppressing the NF-κB pathway by regulating the PPARγ pathway	Hu et al. (2022)
	Mouse EAE model induced by MOG 35-55 and PTX	oral	60 mg/kg	Regulating the Myd88/PI3K/Akt/NF-κB signaling pathway to inhibit microglial activation	Zheng et al. (2022)
	Severe AP model in rats induced by 5.0% sodium taurocholate	oral	40 mg/kg	Inhibiting the NLRP3/IL-1β/CXCL1 signaling pathway to reduce neutrophil infiltration	Xu et al. (2021b)
	Poptosis of H2O2-Induced SH-SY5Y Cells	indicated	10, 20, 50, and 100 μM	Inhibiting the PI3K/mTOR/GSK3β signaling pathway	Li et al. (2020a)
	LPS-induced acute lung injury (ALI) in rats	oral	20 mg/kg, 40 mg/kg	Inhibiting the mTOR/HIF-1α/VEGF signaling pathway	Li et al. (2020b)
	Ovalbumin (OVA) sensitization and challenge to establish mouse bronchial asthma model	i.p	15, 30, 60 mg/kg	Suppressing the Notch signaling pathway	Hua et al. (2019)
	H9c2 cells were treated with LPS	indicated	20 μM	Downregulating miR-223 to inhibit the Jnk signaling pathway	Yang et al. (2019)
	Rat sepsis model induced by cecal ligation and puncture	i.p	10 mg/kg	Activating the JAK1/STAT3 signaling pathway	Chen et al. (2016)
	The NRK-52E cells were incubated with LPS	indicated	20 μM and 40 µM	Inhibiting the TLR2-mediated NF-κB signal pathway	Li et al. (2015b)
	Rat peritoneal macrophages were treated with millimolar ATP	indicated	0.1, 0.3, 1, 3, 10 µM	Inhibiting the activation of P2X₇R	Zhu et al. (2014)
	Oxidative fish oil-induced oxidative stress model of fish *M. amblycephala*	oral	30 mg/kg	Modulating Notch-Nrf2 interaction to improve oxidative stress	Zhu et al. (2014)
Anti-tumor	Mouse liver metastasis model induced by breast cancer cells	indicated	50 µM	Regulateincg AKT and ERK signaling	Li et al. (2023)
	human prostate cancer PC3 and DU145 cells	indicated	1 μM, 5 µM	Regulation of HSP90/MLKL/PGAM signaling through mitochondrial fission	Zhou et al. (2023c)
	GEM-resistant SW1990 cells (SW1990/GZ) were established by doubling the concentration of GEM	i.h	1 mg/kg	Regulating EMT progression	Wei et al. (2023)
	The human hepatocellular carcinoma	indicated	40 μM	Mediating cellular autophagy through the miR-371a-5p/PTEN axis	Wu et al. (2022)
	The human liver cancer cell lines HepG2	incubated	60 μM	Inhibiting the PI3K/AKT/mTOR and Wnt/β-catenin signaling pathways	Qin et al. (2022)
	Mouse model of subcutaneous xenografts of SK-OV-3 tumors	i.p	20 and 40 mg/kg	Inhibiting EMT target	Long et al. (2022)
	NSCLC cell line	incubated	10, 20, 40, 60 μM	Inhibiting the expression of sPLA2-IIa and NF-κB pathways	Zhang et al. (2022a)
	Cell lines TPC-1 cells	incubated	5 and 50 μM	Activating the AMPK pathway	Li et al. (2021a)
	A549 cells	incubated	2, 5, 10 and 20 μM	Inhibiting the expression of Pgp	Peng et al. (2021)
	Renal carcinoma cells	incubated	25, 50, 100 μM	Inhibiting the PI3K/AKT signaling pathway	Wang et al. (2021)
	NSCLC cells	incubated	25, 50, 75 μM	Inhibiting the expression of MTH1	Wahi et al. (2021)
	Pancreatic cancer cell lines	incubated	40 μM	Inhibiting the NF-κB signaling pathway and P-gp function	Tong et al. (2020)
	Human CoCa and colon epithelial cells	incubated	1 ∼ 18 μM and 201 ∼ 15 μM	Inhibiting the MAPK/JNK, PI3K/AKT, NF-κβ, and STAT signaling pathways	Saunders et al. (2019)
	Human ATC cell lines 8505c and SW1736	incubated	10, 15, 20,25 μM	Inhibiting the TRAF6/HIF-1α/VEGF and TRAF6/CD147/MMP9 signaling pathways	Shi and Zhou (2018)
Antibacterial	*S. aureus* CMCC26003	incubated	4 μg/ml,8 μg/ml	Interfering with the release of extracellular DNA and inhibiting the expression of biofilm-related genes	Yan et al. (2017)
	*Staphylococcus aureus* strains	incubated	1.56–200 μg/ml	Regulating the expression of strain-specific genes	Đukanović et al. (2022)
	The rat pneumonia model was established by aspirating *Streptococcus* pneumo_x0002_niae through the trachea	i.p	20 and 80 mg/kg	Decreasing the NF-κB and capsular polysaccharide-protein	Zhou et al. (2023a)
	A mouse model of diarrhea induced by *E. coli* O1	oral	8.75, 17.5 and 35 mg/kg	Increasing the abundance of beneficial intestinal microbiota and inhibiting the abundance of harmful bacteria	Gao et al. (2021)
	Ulcerative colitis was induced by 3% sodium dextran sulfate (DSS) in mice	oral	20 mg/kg	Activating the PPARγ signal pathway	Luo et al. (2022)

**TABLE 3:** Molecular mechanisms to the pharmacological activity of emodin in the treatment of metabolic diseases.

Disease	Models	Dosing method	Dosage of administration	Molecular mechanisms	References
Obesity and hyperlipidemia	C57BL/6J mice were fed a high-fat diet to induce obesity	oral	40, 80 mg/kg	Promoting browning in scWAT and activating the BAT activities	Cheng et al. (2021)
	High-fat diet-induced obese mice	oral	80 mg/kg	Regulating SREBP target gene	Li et al. (2016a)
	Adipose tissue of diet-induced obese rats	external use	4 g/kg	Downregulating the expression of G0S2 protein	Lu et al. (2014)
	Differentiated 3T3-L1 cells	indicated	5, 10, 15, 20 μg/mL	Inhibiting the activity of 11β- HSD1 activity	Yu et al. (2020a)
	3T3-L1 preadipocyte cells	indicated	80 μM	Inhibiting pancreatic lipase	(Guo et al., 2014)
NAFLD	High-fat diet-induced NAFLD mice	oral	80, 40	Inhibiting FXR expression	Shen et al. (2021)
	Egg yolk powder-induced NAFLD zebrafish	oral	0.5, 0.25 μg/mL	Reducing hepatic lipogenesis by activating AMPK signaling	Yu et al. (2020b)
	HFD-induced obese rats	oral	80, 40 mg/kg	Attenuating lipid accumulation by activation of the AMPK signaling pathway	Tzeng, et al. (2012a)
	Liquid fructose-induced NAFLD rat	oral	160, 80, 40 mg/kg	Improving the lipid accumulation through the ERS-SREBP1c pathway	Li et al. (2016b)
	Moderate to severe hepatocyte steatosis model in rat	oral	40 mg/kg	Increasing the expression of hepatic PPAR gamma mRNA	Dong et al. (2005)
	LPS-induced macrophage inflammation model	indicated	25 mM	Reducing macrophage expression of pro-inflammatory cytokines by suppressing Erk1/2 and p38 signaling pathways	Jia et al. (2014)
	LPS-induced liver injury model in mice	oral	50 mg/kg	Suppressing NF-κB pathway	Xie et al. (2019)
	APAP-induced liver toxicity model in mice	oral	10, 30 mg/kg	Inhibiting oxidative stress in the liver via AMPK with Hippo/Yap signaling pathway	Lee et al. (2020)
DM and its complications	DIO mice	oral	100 mg/kg	Decreasing 11β-HSD1 activity	Feng et al. (2010)
	The genetic type 2 diabetic ob/ob mice	i.p	50 mg/kg	Decreasing 11β-HSD1 activity and increasing adiponectin and PPARγ mRNA levels	Wang et al. (2012)
	L6 myotubes	indicated	3 μM	Activating AMPK by inhibiting mitochondrial respiratory complex I activity	Song et al. (2013)
	Streptozotocin (STZ)-induced diabetic mice	i.p	1.5 mg/kg	Activating PPARγ	Xue et al. (2010)
	Injury of HUVECs caused by high concentration of glucose	indicated	1, 3, 10, 30 mM	Protecting endothelial cells by activating MAPK signaling pathways	Gao et al. (2015)
	STZ-induced diabetic rat model	oral	20 and 40 mg/kg	Reducing proteinuria and alleviating renal fibrosis via the AMPK/mTOR pathway	Liu et al. (2021)
	KK-Ay mice model of DN	oral	40 and 80 mg/kg	Inhibiting the PERK/eIF2α axis	Tian et al. (2018)
	Mesangial cells cultured by high-glucose	indicated	40, 20 μM	Suppressing TGF-β1 and FN overexpression through inhibition of NF-κB activation	Yang et al. (2013)
	The rat mesangial cell line (HBZY-1) was cultured in a glucose condition	indicated	30, 60 μM	Inhibiting the p38MAPK signaling pathway	Li et al. (2009)
	Streptozotocin-induced diabetic rat with nephropathy model	oral	40 mg/kg	Ameliorating renal dysfunction by inhibiting the activation of the p38 MAPK pathway	Wang et al. (2006)
	Type 2 DM rat model induced by high fat chow and low dose STZ injection	oral	100, 50 mg/kg	Regulating Akt/GSK-3β signaling pathway	Wu et al. (2014)
OP	IBD-induced osteoporosis rat model	oral	30 mg/kg	Suppressing osteoporosis by inhibiting osteoclast formation	Luo et al. (2020)
	Osteoclasts of bone marrow cells (BMCs) treated with M-CSF and RANKL	indicated	10 μM	Enhancing osteoblast differentiation and bone growth and suppressing osteoclast differentiation and bone resorption	Kim et al. (2014)
	BMSCs	indicated	0.1 and 10 μM	Inhibiting adipocyte differentiation and enhancing osteoblast differentiation	Yang et al. (2014)
	Murine pre-osteoblastic MC3T3-E1 cells were pre-treated with Noggin	indicated	5 μM	Regulating BMP-9 expression and phosphorylation of p38	(Cheng et al., 2017)
	Mouse osteoblastic MC3T3-E1 subclone 4 cells	indicated	5 mM	Upregulation of bone morphogenetic protein (BMP) through the activation of both Akt and MAP kinases by activating PI3K	Lee et al. (2008)

## 4 Metabolic diseases treated with emodin

**FIGURE 2:**
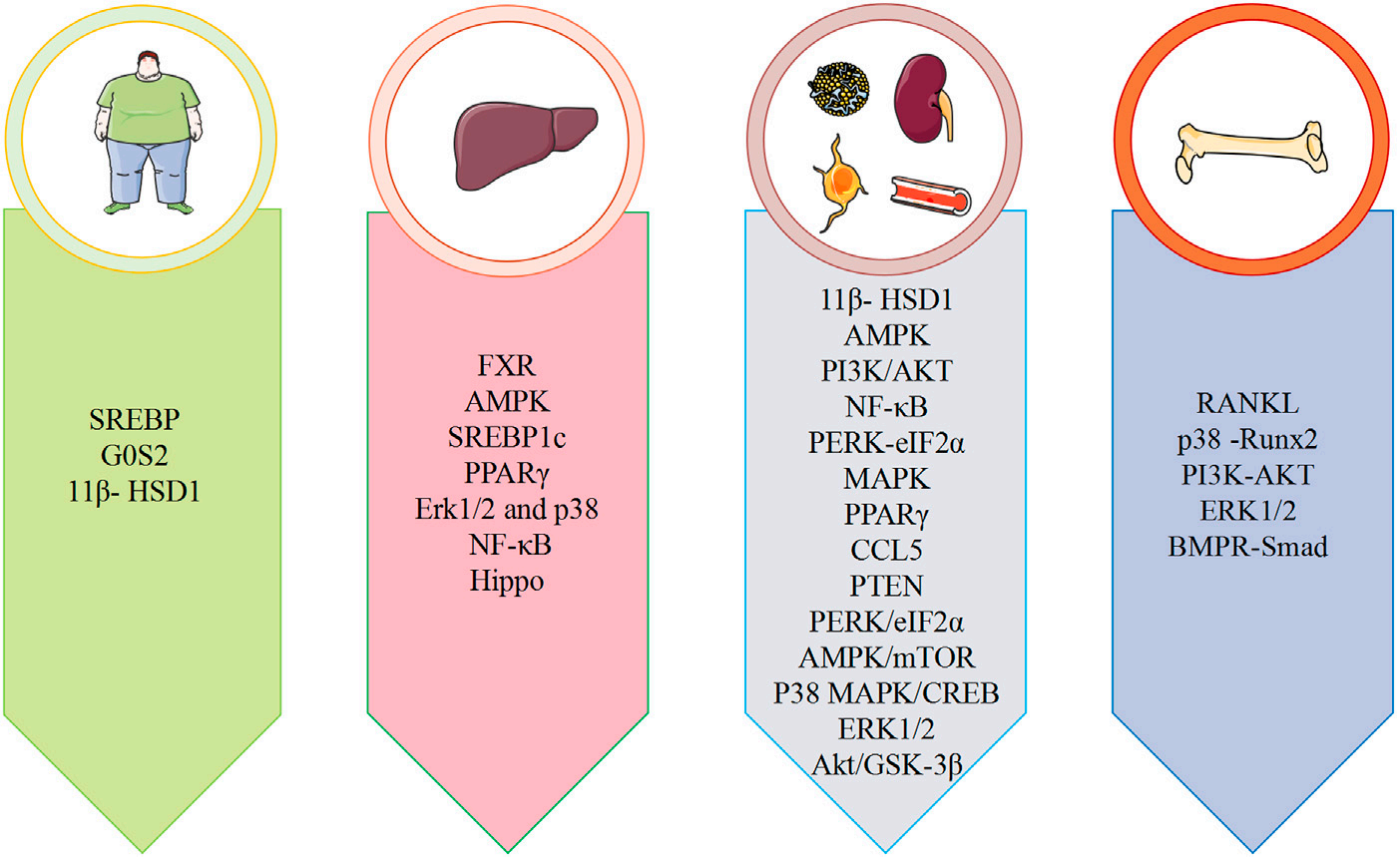
Summary of pharmacological effects of emodin in the treatment of metabolic diseases.

**FIGURE 3:**
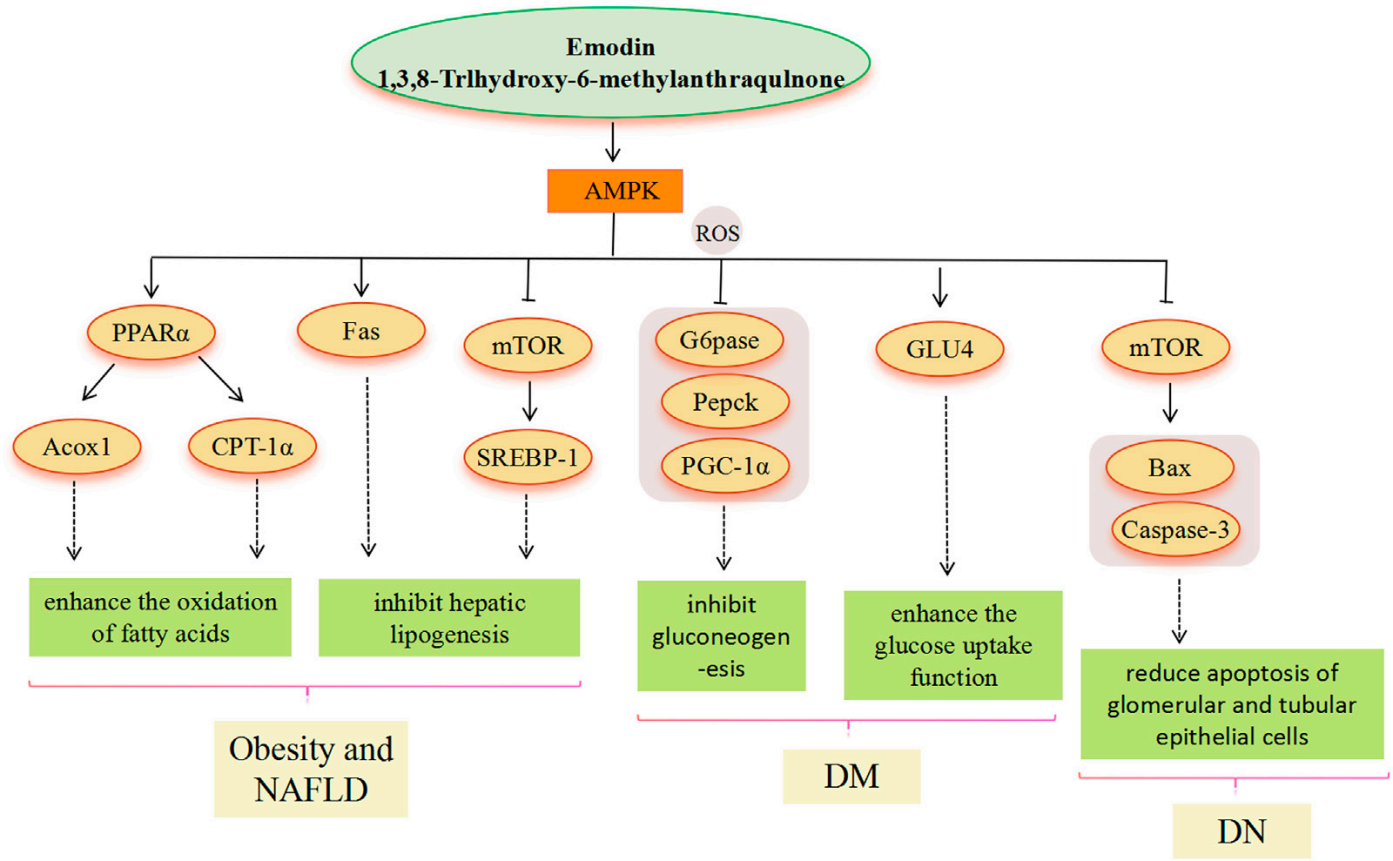
AMPK signaling pathway involved in the pharmacological actoin of emodin in the treatment of metabolic diseases.

**FIGURE 4:**
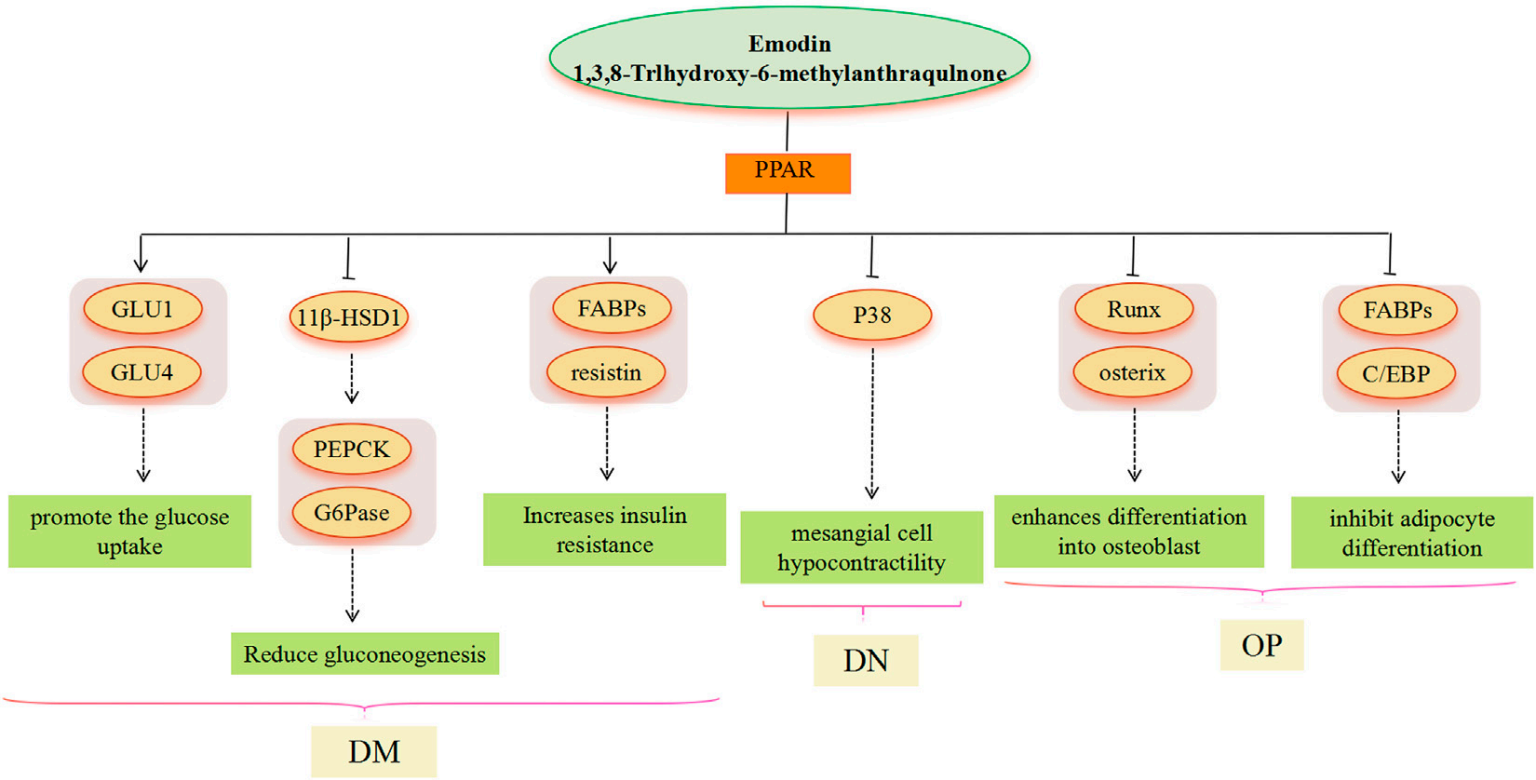
PPAR signaling pathway involved in the pharmacological actoin of emodin in the treatment of metabolic diseases.

**FIGURE 5:**
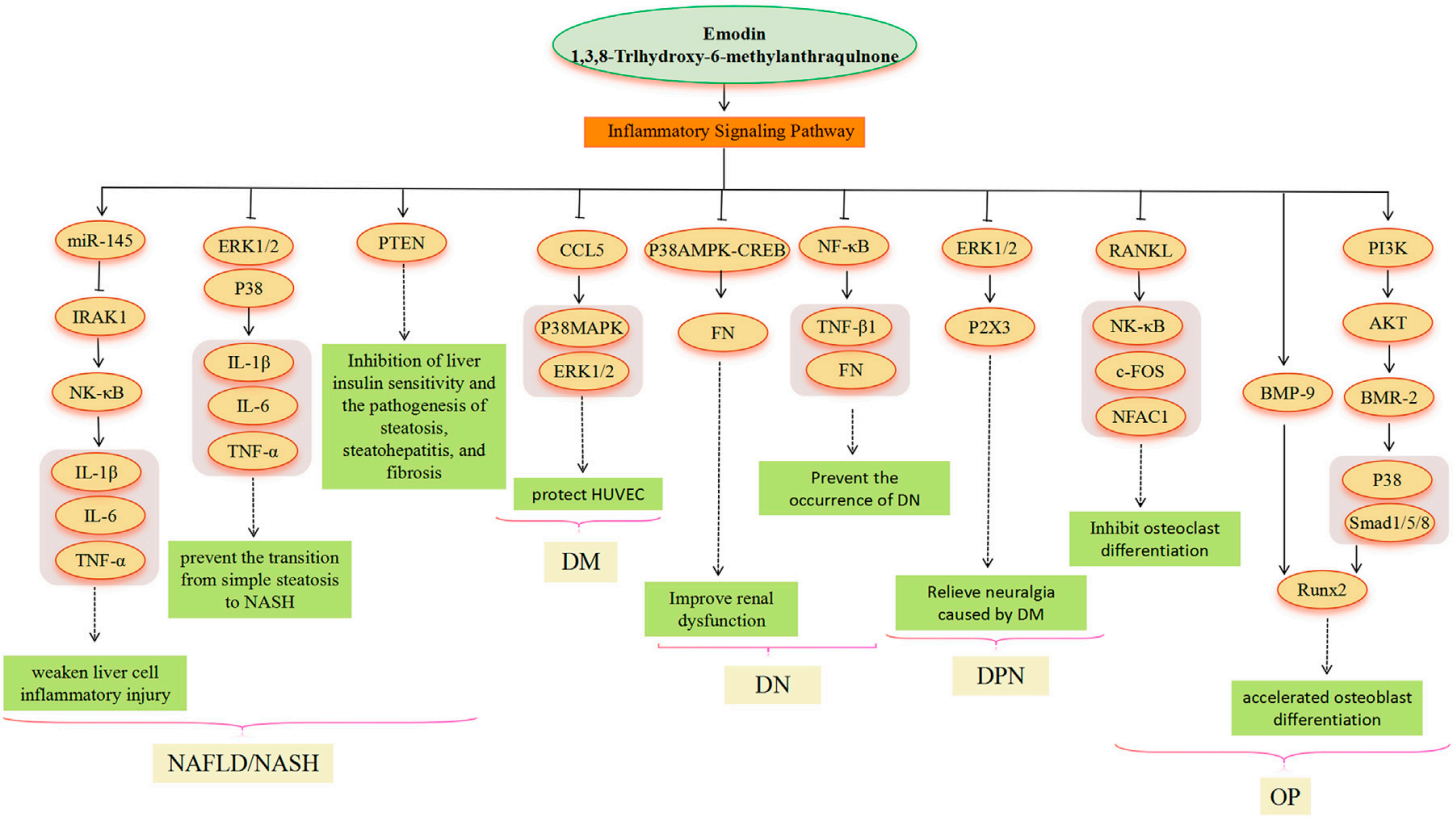
Inflammatory signaling pathway involved in the pharmacological actoin of emodin in the treatment of metabolic diseases.

Metabolic diseases are caused by a variety of reasons, and the pathogenesis is very complex. The beneficial effects of emodin on metabolic diseases have been confirmed by several studies. We summarized several studies and found that emodin has a good therapeutic effect on obesity, hyperlipidemia, hepatocyte steatosis, diabetes and its complications, and osteoporosis. The pharmacological molecular pathways of emodin in treating metabolic diseases are shown in Table 3. The main targets of action of emodin in the treatment of metabolic diseases are shown in Figure 2. The molecular pathways involved in the pharmacological action of emodin in the treatment of metabolic diseases are presented in Figures 3–5.

### 4.1 Obesity and hyperlipidemia

Obesity caused by energy imbalance, which is associated with hypertrophy and hyperplasia of adipocytes, is increasingly affecting the health of all human beings (Muir et al., 2016). The molecular biology of the energy imbalance is reflected in the production of more fat than the decomposition of fat (Unger and Orci, 2001). Under obese status, hypertrophic adipocytes will release higher levels of free fatty acids (FFA) (Su and Peng, 2020). It suggests that obesity is related to a disorder of lipid metabolism; there is evidence that 43.2% of those who are obese live with hyperlipidemia (de Sereday et al., 2004). Adipose tissue plays an essential role in modulating energy metabolism (Song et al., 2017). Activating brown adipose tissue (BAT) and inducing browning in white adipose tissue (WAT) has been suggested as one of the important ways for obesity treatment (Thyagarajan and Foster, 2017; Kuryłowicz and Puzianowska-Kuźnicka, 2020). In C57BL/6J mice that were fed a high-fat diet to induce obesity, emodin (40 mg/kg, 80 mg/kg) reduced the body weight and food intake of obese mice. The mechanism of emodin improving obesity was related to the promotion of scWAT browning and activation of BAT activity (Cheng et al., 2021). Similarly, in high-fat diet-induced obese mice, treatment with emodin (80 mg/kg) reduced body weight, lowered blood lipids, liver cholesterol, and triglyceride levels, and reduced the size of white and brown fat cells, probably due to the regulation of sterol-regulatory element binding proteins (SREBPs) target genes (Li J. et al., 2016). Other studies have shown that the improvement of obesity by emodin was related to the expression of the G0/G1 switch gene 2 (G0S2) protein in adipose tissue, which plays an important role in energy metabolism in the organism (Lu et al., 2014). 11β-hydroxysteroid dehydrogenase type 1 (11β-HSD1) in adipose tissue is also an effective therapeutic target for obesity. In Ap-EMO (5–20 μg/mL) incubated differentiated 3T3-L1 cells, the 11β- HSD1 activity and protein expression decreased in a dose-dependent manner (Yu B. et al., 2020). Moreover, pancreatic lipase is the most important enzyme for the hydrolysis of dietary fat, and alterations in its expression are closely associated with metabolic diseases such as obesity (Bessesen and Van Gaal, 2018). In the 3T3-L1 preadipocyte cells, enhanced inhibition of pancreatic lipase was observed when emodin was combined with apigenin and naringenin, further demonstrating that emodin (80 μM) may act as a synergistic agent for the prevention of obesity (Guo et al., 2016). Therefore, emodin can become a preventive and therapeutic drug for obesity by regulating energy metabolism and lipid metabolism.

### 4.2 Non-alcoholic fatty liver disease

Alongside the global increase in metabolic syndrome, a sharp rise has been seen in the prevalence of non-alcoholic fatty liver disease (NAFLD), which is predicted to become the most frequent indication for liver transplantation by 2030 (Byrne and Targher, 2015). NAFLD covers the progression from simple steatosis to non-alcoholic steatohepatitis (NASH) and eventually to cirrhosis and hepatocellular carcinoma (Lau and Wong, 2018). Modern research has shown that the “multiple-hit” hypothesis has emerged as the main pathogenesis of NAFLD (Buzzetti et al., 2016), including the metabolism of fatty acids, endoplasmic reticulum stress (ERS), insulin resistance (IR), and inflammasome activation (Friedman et al., 2018). Recently, a large number of experimental studies have reported that emodin has therapeutic effects on various fatty liver models through different molecular mechanisms. Emodin is a putative Farnesoid X receptor (FXR) activator. In an HFD-induced NAFLD model in mice, emodin (40, 20, and 80 mg/kg) improved HFD-induced liver lipid accumulation, IR, inflammation, and oxidative stress by activating FXR (Shen et al., 2021). Moreover, in an egg yolk powder-induced NAFLD model in zebrafish, emodin (0.5 and 0.25 μg/mL) significantly reduced the levels of TG, TC, and NEFA in NAFLD zebrafish. Furthermore, emodin reduced hepatic lipogenesis by upregulating the expressions of lipid metabolism-related genes by activating AMPK signaling, such as Phosphatidylinositol 3-kinase (PI3K), AKT serine/threonine kinase 2 (AKT2), PPARα, carnitine palmitoyl transferase 1α (CPT-1α), and peroxisomal acyl-CoA oxidase1 (ACOX1) (Yu L. et al., 2020). Similarly, emodin enhanced the phosphorylation of AMPK and its primary downstream targeting enzyme, Acetyl coenzyme A (acetyl-CoA) carboxylase1 (ACC1), upregulated the expression of CPT-1 protein, and downregulated SREBP1 and Fas protein levels in hepatocytes of HFD-fed rats (Tzeng et al., 2012b). Another study suggested that the downregulation of emodin on SREBP1 might be related to the AMP-activated protein kinase/mammalian target of rapamycin (AMPK/mTOR) axis (Wang S. et al., 2017). In addition, emodin (160, 80, and 40 mg/kg) improved hepatic steatosis by inhibiting the activation of SREBP1c and its target genes in the liver tissue of fructose-fed rats and increasing the expression of CAP-1. Furthermore, the activation of SREBP1c was related to emodin regulation of ERS-related protein expression (Li X. et al., 2016). PPARγ is also thought to be involved in the treatment of fatty liver disease. In moderate to severe hepatocyte steatosis model in rats, emodin (40 mg/kg) improved liver lipid deposition and histological structure. This therapeutic mechanism might be related to the fact that emodin enhanced the expression of hepatic PPARγ mRNA (Guo et al., 2016).

In addition, emodin can also prevent the transition from NAFLD to NASH by regulating inflammation signaling pathways. In the mice model where a combination of HPD and LPS caused NAFLD to develop into NASH, emodin (40 mg/kg) significantly attenuated the functional damage and inflammatory response of the liver. Authors then performed *in vitro* experiments to examine whether emodin could directly inhibit the LPS-induced inflammatory response in macrophages, and the result showed that emodin (25 mM) reduced macrophage expression of pro-inflammatory cytokines tumor necrosis factor-α (TNF-α), Interleukin-1β (IL-1β), and Interleukin-6 (IL-6), as well as iNOS. It was also shown that emodin inhibited LPS-induced inflammation in macrophages by inhibiting the extracellular signal-regulated kinase (Erk1/2) and p38 mitogen-activated protein kinase (p38 MAPK) signaling pathways (Jia et al., 2014). Similarly, in an LPS-induced liver injury model in which mice were pre-treated by the oral administration of emodin (50 mg/kg), a reduction in the expression of LPS-triggered pro-inflammatory factors (IL-1β, IL-6, and TNF-α) and downregulation of serum alanine transaminase (ALT) and aspartate transaminase (AST) levels were observed. The therapeutic mechanism might be related to the reduction of nuclear factor-kappaB (NF-κB) pathway-mediated miR-145 and the enhancement of IL-1 receptor-associated kinase 1 (IRAK1) in liver tissues (Xie et al., 2019). In addition, in an APAP-induced liver toxicity model in mice, emodin (10 mg/kg) could normalize the histological structure of the liver in the pretreatment group. In the quantitative RNA-seq analysis using RNA samples from liver tissues, several of the most strongly induced genes were targets of the AMPK signaling pathway and the Hippo signaling pathway (Lee et al., 2020). All these findings suggest that emodin is a promising agent for preventing dietary induction of NAFLD and preventing progression to NASH and that it is mainly related to the inhibition of hepatic lipid accumulation, improvement of lipid oxidative stress and ERS, and amelioration of inflammation with the involvement of multimolecular targets.

### 4.3 Diabetes mellitus and its complications

Diabetes mellitus (DM) is a metabolic disease characterized by hyperglycemia, which is caused by defective or impaired biological action of insulin secretion (Martorell et al., 2021). Type 1 diabetes is characterized by the destruction of pancreatic β-cells, while defective insulin secretion is a feature of type 2 diabetes (Forbes and Cooper, 2013). The factors affecting insulin secretion and insulin action include glucotoxicity, lipotoxicity, and nutri-toxicity (Solis-Herrera et al., 2000; Pietropaolo and Le Roith, 2001). Oxidative stress can lead to inflammation, IR, dyslipidemia, hyperglycemia, and other abnormalities. It is an important mechanism for obesity to cause IR and impaired insulin secretion, and is also an important factor in the subsequent development of DM (Rochette et al., 2014). The oxidative stress effect leads to DM complications, and the resulting complications are grouped under “microvascular disease (retinopathy, nephropathy and neuropathy)” and “macrovascular disease (atherosclerosis, coronary artery disease, cerebral thrombosis, gangrene of the limbs, and renal arteriosclerosis)” (Forbes and Cooper, 2013).

Many studies have confirmed that emodin has a good therapeutic effect on type 2 diabetes. 11β-HSD1 may be one of the main emodin targets. 11β-HSD1 is the major reductase in most intact cells and catalyzes the regeneration of active glucocorticoids, which are selectively elevated primarily in the adipose tissue of patients who are obese and lead to metabolic disorders including IR, central obesity, hyperglycemia, and dyslipidemia. In DIO mice, emodin (50 and 100 mg/kg) significantly lowered blood glucose and increased insulin sensitivity. Furthermore, emodin (100 mg/kg) decreased the serum concentration of TG and TC and reduced the level of hepatic phosphoenolpyruvate carboxykinase (PEPCK) and glucose-6-phosphatase-α (G6Pase-α) mRNA; its mechanism of action was related to 11β-HSD1 activity. The 11β-HSD1 activity in liver and mesenteric adipose tissue treated with emodin (100 mg/kg) was significantly reduced by 53.5% and 41.2%, respectively (Feng et al., 2010). Similarly, in the genetic type 2 diabetic ob/ob mice, the activity of 11β-HSD1 was significantly decreased in the mesenteric adipose tissue of mice treated with emodin (50 mg/kg). The random-fed and fasting plasma glucose levels of diabetic rats treated with emodin (50 mg/kg) for a long period were significantly lower, which might be related to the significant increase in adiponectin and PPARγ mRNA levels caused by emodin (Wang et al., 2012). There is evidence that adiponectin and PPARγ are related to the activity of 11β-HSD1 (Chinetti-Gbaguidi et al., 2012), therefore, the inhibition of 11β-HSD1 in adipose tissue caused by emodin is expected to improve adipocyte dysfunction and play a beneficial role in type 2 diabetes or metabolic diseases. The activation of AMPK by emodin is also one of the ways to treat DM. Studies have reported that administration of emodin (1 mg/kg) significantly reduced fasting plasma glucose levels and improved high fat-induced glucose tolerance in DM mice. More importantly, the studies found that emodin (3 μM) enhanced glucose transporter 4 (GLUT4) translocation and [(14)C]glucose uptake into the myotube in an AMPK-dependent manner in L6 myotubes. In addition, the reduction of glucose production in the body caused by emodin was associated with the inhibition of the expression of key gluconeogenesis-related genes (PEPCK and G6Pase-α) in hepatocytes. This inhibitory effect on glucose production was associated with emodin’s activation of AMPK by inhibiting the activity of mitochondrial respiratory complex I, resulting in increased reactive oxygen species and Ca (2+)/calmodulin-dependent protein kinase kinase (CaM-KK) activity (Song et al., 2013). Martorell et al. suggested that the therapeutic effects of emodin on diabetes include reducing the production of inflammatory cytokines, increasing insulin secretion, and improving insulin sensitivity (Martorell et al., 2021). In a study, emodin (1.5 mg/kg) significantly increased the expressions of PPARγ and LPL and decreased the expression of fatty acid translocase (FAT/CD36) in the liver of diabetic mice. Emodin also had effects on the gene expression in the subcutaneous adipocyte tissue, elevated the expression of fatty acid binding proteins (FABPs/ap2), and reduced the expression of resistin. FABPs (ap2) have been commonly used to evaluate the activity of PPARγ ligands (Xue et al., 2010). Emodin can also mediate diabetes through the inflammatory signaling pathway. The enhanced adhesion of monocytes to human umbilical vein endothelial cells (HUVECs) and elevated chemotaxis activities in HUVECs cultured in high glucose medium were completely reversed by emodin. Emodin (1, 3, 10, and 30 mM) protected endothelial cells in a high glucose environment by inhibiting the inflammatory response and subsequent apoptosis generated by the MAPK signaling pathway involved in chemokine (C-C Motif) Ligand 5 (CCL5) (Gao et al., 2015).

Emodin also has a therapeutic effect on complications of DM. There are many research reports about emodin on diabetes nephropathy (DN), and the treatment is mostly related to inflammatory mechanisms. Research has demonstrated that emodin (20 and 40 mg/kg) reduced proteinuria and attenuated nephrogenic fibrosis in DN rats, and the mechanism of nephroprotective effect was related to the inhibition of apoptosis through the AMPK/mTOR signaling pathway and regulation of autophagy-related protein expression, including LC3-II/I, Beclin-1, p-AMPK, p62, and p-mTOR (Liu et al., 2021). In a KK-Ay mice model of DN, the results showed that emodin (40 and 80 mg/kg) treatment ameliorated urine albumin, serum creatinine, and blood urea nitrogen of DN mice. Tian et al. showed that the therapeutic mechanism of emodin and its inhibition of the PKR-like ER kinase/Eukaryotic initiation factor 2α (PERK/eIF2α) axis and improvement of podocyte apoptosis were induced by ER stress (Tian et al., 2018). Under high glucose conditions, it is well known that the production of tumor necrosis factor-β1 (TNF-β1) and fibronectin (FN) in mesangial cells increases. In HG-incubated mesangial cells, emodin (20, 40 μM) could mediate the production of TNF-β1 and FN through the NF-κB signaling pathway (Yang et al., 2013). Recent studies have identified high-glucose-induced p38 MAPK (p38) over-activation in mesangial cells. Studies have shown that reducing the contractile force of mesangial cells is the main potential mechanism of emodin intervention in DN. The rat glomerular mesangial cells (HBZY-1) were cultured in glucose condition, and emodin (30 and 60 μM) significantly suppressed HG-induced cell proliferation and arrested cell cycle progress, which is related to the p38MAPK pathway (Li et al., 2009). Moreover, in the streptozotocin-induced diabetic rats with nephropathy, emodin (40 mg/kg) reduced glomerular area, glomerular volume, and related biochemical parameters such as serum creatinine, plasma urea nitrogen, and proteinuria. The mechanism of emodin improving renal function was related to its inhibition of the P38 MAPK signaling pathway and downregulation of FN expression (Wang et al., 2006). This suggests that P38 MAPK is an important target for emodin to participate in DN. In addition, emodin-mediated oxidative stress is also involved in the DN process. Nrf2 acts as an important transcription factor regulating antioxidant stress. The concentration of MDA was significantly increased in the Nrf2 knockout HK-2 cell model, and after the intervention of emodin (40 μM), the expression of Nrf2 increased and the concentration of MAD decreased correspondingly (Ji et al., 2023).

In addition, emodin also has a good therapeutic effect on diabetic neuropathic pain (DNP), diabetic cardiomyopathy (DCM), and diabetic gastroenteropathy. Nano emodin (5 mg/ml) reduced extracellular signal-regulated kinase 1/2 (ERK1/2) phosphorylation in DRG of diabetic rats to alleviate Purin 2X3 (P2X3) receptor-mediated hyperalgesia (Li L. et al., 2017). In a rat model of type 2 DM that was induced by high fat chow and low dose STZ injection, emodin (50 and 100 mg/kg) showed significant improvement of myocardial function and induced a significant increase in phosphorylation of glycogen synthase kinase-3β (GSK-3β) in the myocardium (Wu et al., 2014). Moreover, emodin was able to restore the expression of Cav1.2 cardiac myocytes of hyperlipidaemic-diabetic rats (Zhao et al., 2009). Wang et al. established a diabetic rat gastroenteropathy model with HS/HF combined with STZ, and after treatment with emodin (30, 60, and 90 mg/kg), the gastrointestinal transmission rate and serum substance P (SP) expression were increased, and serum vasoactive intestinal peptide (VIP) expression was decreased (Wang et al., 2023). It is suggested that emodin has a wide therapeutic effect on diabetes and its complications, which involves multiple targets.

### 4.4 Osteoporosis

Osteoporosis (OP) is a systemic bone disease characterized by a reduction in bone mass, a decrease in the number of bone trabeculae, and a deterioration of the microstructure of bone tissue, leading to increased brittleness of bone and susceptibility to fracture (Kanis et al., 1994; Seeman, 2003). In China, emodin has been used for thousands of years to treat bone diseases. However, the underlying mechanism of emodin for OP treatment is still poorly understood. In an LPS-induced bone resorption model in mice, a CT imaging biomarker confirmed that emodin is a potential drug for LPS-mediated OP (Kang et al., 2014). It is known that inflammation, corticosteroid use, vitamin D deficiency, and malnutrition are key factors leading to bone loss caused by inflammatory bowel diseases (IBD) (Schulte et al., 2000; Moschen et al., 2005). Emodin has a good therapeutic effect on OP associated with IBD. In an IBD-induced OP model in rats, emodin (30 mg/kg) could inhibit the formation of osteoclast and reduce the mRNA expressions of tumor necrosis factor receptor-associated factor 6 (TRAF6), nuclear factor of activated T cells, cytoplasmic 1 (NFATc1) and c-fos related to the differentiation of osteoclasts (Luo et al., 2020). Similarly, in an LPS-induced osteoclast formation model in ICR mice and osteoclasts of bone marrow cell (BMC) treated with macrophage-CSF (M-CSF) and RANKL, emodin could be a potential drug for the treatment of OP due to its ability to enhance osteoblast differentiation and bone growth and inhibit osteoclast differentiation and bone resorption (Kim et al., 2014). In addition, another study suggested that emodin could not only regulate the formation of osteoblasts but also participate in lipid generation, achieving a differentiation balance between osteoblasts and adipocytes. In bone marrow stromal cells (BMSCs), emodin (0.1 μM and 10 μM) increased the genes and proteins expression of osteogenesis markers, such as Runx2, Osterix (OSX), Cllagen type I (Col1), Osteocalcin (OC) and alkaline phosphatase (ALP), whereas the genes and proteins involved in adipogenesis were downregulated, such as PPARγ, CCAAT/enhancer binding protein (C/EBPα), and ap2 (Yang et al., 2014). The p38 signaling pathway also plays an important role in the treatment of OP with emodin. Emodin has been shown to enhance osteoblast differentiation and stimulate bone area formation by activating the p38-Runx2 pathways (Kim et al., 2014). In addition, the bone morphogenetic proteins (BMPs) belong to the TGF-β family, which promotes the directed differentiation of mesenchymal cells into osteoblasts and is a major factor in the induction of bone and cartilage formation. In ovariectomized (OVX) rats, emodin (100 mg/kg) combined with a low dose of estrogen reduced bone loss compared with low-dose estrogen alone. In murine pre-osteoblastic MC3T3-E1 cells that were pre-treated with 100 ng/ml Noggin (BMP inhibitor), emodin (5 μM) promoted MC3T3-E1 cell differentiation and mineralization, which is related to BMP-9 expression and phosphorylation of p38 (Chen et al., 2017). Moreover, during differentiation of mouse osteoblast MC3T3-E1 subclone four cells, emodin (5 μM) was found to accelerate osteoblast differentiation by activating PI3K-Akt/MAP kinase and thus inducing BMP-2 gene expression. In summary, the treatment of OP with emodin is mainly related to osteogenesis, adipogenesis, and inflammation (Lee et al., 2008).

In recent years, many studies have shown that metabolic diseases are closely related to intestinal flora disorders, including NAFLD, DM, and OP (Yong et al., 2020; He L. et al., 2022; Liu J. et al., 2022; Tian et al., 2023). Emodin has the effect of regulating intestinal flora, but it is mainly used in research on enhancing immunity and anti-inflammatory activity (Zhang M. et al., 2022; Mabwi et al., 2023). Therefore, it is recommended that the intervention of intestinal flora should be considered as a research direction for emodin to treat metabolic diseases.

## 5 Molecular mechanism

### 5.1 AMPK signaling pathway

AMPK is a heterotrimer (composed of catalytic α and regulatory β and γ subunits) and activation of AMPK can maintain energy homeostasis by decreasing anabolism and increasing catabolism. AMPK is highly valued as a potential target for the treatment of metabolic diseases. As an AMPK agonist, the main pathway of emodin in the treatment of metabolic diseases includes the AMPK signal pathway (Park et al., 2018). Through the above literature search, we found that the metabolic diseases involved in the AMPK signal pathway mainly include obesity, NAFLD, and DM. In the treatment of obesity and hyperlipidemia, regulation of the expression of downstream genes related to lipid metabolism based on emodin-activated AMPK targets is the main modality. The enhancement of AMPK activity, inhibition of ACC1 activation, and upregulated CPT-1 gene expression suggest that emodin may promote hepatic fatty acid oxidation to reduce lipid accumulation by activating AMPK. Emodin is able to activate AMPK to suppress the HFD-induced increase in gene expression of SREBP-1 and FAS, which ultimately inhibits adipogenesis (Tzeng et al., 2012a). Similarly, in the treatment of NAFLD, emodin can also increase mitochondrial fatty acid β-oxidation by mediating AMPK activation to reduce hepatic lipid accumulation, the molecular mechanism involves the improvement of IR in the PI3K/AKT2/AMPKα pathway and the promotion of gene expression of PPARα, CPT-1a and ACOX1 related to fatty acid oxidation (Tzeng et al., 2012b; Yu L. et al., 2020). Moreover, stimulation of AMPK activation by emodin may prevent the translocation of SREBP-1 to the nucleus, thereby inhibiting hepatic adipogenesis through the downregulation of SREBP-1 and FAS expression (Tzeng et al., 2012b). In addition, Wang et al. suggested that SREBP-1 inhibition was associated with emodin inhibition of the AMPK-MTOR-related autophagic pathway via Camkk (Wang et al., 2016). Emodin can prevent and treat the occurrence and development of NAFLD from IR and oxidative stress of lipid deposition by activating AMPK (Hu et al., 2020). In the treatment of DM, AMPK is required for the organism to maintain glucose homeostasis and is central to insulin sensitivity in muscle, especially in post-exercise glucose uptake and subsequent glycogen resynthesis (Ovens et al., 2021). Emodin phosphorylates AMPK and inhibits gluconeogenesis in hepatocytes, resulting in relative changes in gluconeogenesis levels, for example, G6pase, Pepck, and peroxisome proliferator-activated receptor-γ coactivator-1α (PGC-1α) (Song et al., 2013), which lowered blood glucose (Li X. et al., 2015). In addition, emodin can activate adiponectin and IRS-1, and then activate the AMPK signal pathway, thereby promoting GLU4 protein expression and enhancing glucose uptake in adipocytes (Yang et al., 2007; McGee et al., 2008). In the treatment of DN, AMPK phosphorylation is inhibited in renal tissues with DN, suggesting that AMPK phosphorylation may have a preventive role against DN (Lee et al., 2010; Mihaylova and Shaw, 2011). Emodin upregulates AMPK phosphorylation, downregulates mTOR phosphorylation, and reduces the expression of Bcl-2-associated X protein (Bax) and cysteine-dependent aspartate-specific proteases-3 (caspase-3), indicating that emodin actively regulates autophagy, thereby reducing apoptosis of glomerular and tubular epithelial cells (Liu et al., 2021).

### 5.2 PPAR signaling pathway

PPARs are a class of nuclear receptors that regulate lipid metabolism and glucose metabolism by binding to steroids, triglycerides, and glucose. Modern research has found that PPAR agonists have important metabolic regulatory effects and PPAR agonists, such as thiazolidinediones, fibrates, and gliclazides, are now approved as clinical agents for the treatment of dyslipidemia and DM (Iglesias and Díez, 2006). In the treatment of NAFLD, PPAR overexpression is a general property of steatotic livers, but it seems more likely to affect liver lipid deposition by regulating PPAR and then acting on SREBP. In the treatment of DM, adipose cells are a major target tissue for PPARγ agonists (Skopková et al., 2007). Emodin, with PPARc ligand-binding activity, greatly promotes the glucose uptake and differentiation in adipocytes (Yang et al., 2007), thereby inducing an increase in glucose uptake in a time-and dose-dependent manner, as well as GLUT1 and GLUT4 mRNA expression, and GLUT1 and GLUT4 vesicles in adipocytes as a PPARg ligand (Yang et al., 2007; Choi et al., 2011). Furthermore, emodin activates PPAR, thus affecting the expression of downstream genes FABPs (ap2) and resistin, among which, ap2 plays a critical role in dyslipidemia, IR, and atherosclerosis in humans. However, resistin is controversial in obesity, IR, and glucose homeostasis (Steppan et al., 2001; Janke et al., 2002; Lee et al., 2003; Xue et al., 2010). 11β-HSD1 seems to play an important role in the treatment of DM with emodin, and the administration of emodin significantly reduces hepatic concentrations of PEPCK and G6Pase mRNA, which is consistent with observations in 11β-HSD1 knockout mice. This suggests that PEPCK and G6Pase may be increased by 11β-HSD1 to catalyze the rate-limiting steps of gluconeogenesis (Alberts et al., 2003). There is evidence that 11β-HSD1 gene expression is induced by PPARγ activation (Chinetti-Gbaguidi et al., 2012). Emodin activates PPAR, which is also involved in inflammation-related pathways. In the treatment of DN, emodin effectively ameliorates p38 overactivation via activation of PPARγ (Liu et al., 2009), thereby inhibiting renal mesangial cells’ hypocontractility under a high glucose environment. In the treatment of OP, age-related OP is characterized by reduced bone mass and lipid accumulation in the bone marrow, which results from an imbalance between osteogenic and lipogenic differentiation of bone marrow mesenchymal stem cells (Wen et al., 2023). Adipocyte differentiation is under the control of PPARs. Emodin inhibits adipocyte differentiation by reducing the expression of ap2 and C/EBP proteins related to lipogenesis, which may be related to the PPAR signaling pathway (El-Jack et al., 1999). Studies have shown that the expression of ap2 and C/EBP is mediated through PPARγ activation (Pelton et al., 1999; Zhao et al., 2013). Most current research results indicate that emodin has the effect of activating PPARγ in a dose-dependent manner (Fu et al., 2014), however, the research results of Yang et al. showed that emodin can reduce the expression of PPARγ and enhance the expression of Runx2 and OSX, thereby enhancing the differentiation into osteoblasts (Yang et al., 2014). Runx2 is a master transcription factor regulating both embryonic bone development and postnatal osteoblastic function, and OSX acts downstream of Runx2/core binding factor α 1 (Cbfa1) (Ducy et al., 1999; Nakashima et al., 2002); both play an important role in the cellular decision to initiate differentiation into osteoblasts (Komori, 2003).

### 5.3 Inflammatory signaling pathway

An inflammatory reaction is involved in the whole process of obesity, NAFLD, DM and its complications, and OP (Mundy, 2007; King, 2008; Duarte et al., 2015). Emodin has a good anti-inflammatory effect, which involves multiple signal pathways (Lu et al., 2013; Pang et al., 2015). In the treatment of NAFLD, chronic liver inflammation plays a crucial role in promoting the transition from simple steatosis to NASH and beyond. It contributes to the progression of NASH through low but sustained production of pro-inflammatory cytokines that persistently impair immune monitoring of the liver (Jia et al., 2014). LPS molecules bind to receptors on the surface of the organism’s immune cells, thereby activating the immune cells and causing an inflammatory response. The research found that emodin significantly inhibited the activation of ERK1/2 and P38 by LPS in macrophages, suggesting that emodin may be able to improve systemic inflammation, reduce liver inflammatory cell infiltration, and alleviate liver function damage (Jia et al., 2014). In addition, emodin attenuates LPS-triggered inflammatory damage in hepatocytes *in vitro* and *in vivo* by increasing miR-145, decreasing IRAK1, and then inhibiting the NF-κB pathway (Xie et al., 2019). Dysregulated phosphatase and tensin homolog (PTEN) expression and/or activity have also been associated with the development of several liver diseases. Dysregulated PTEN expression/activity has been observed in obesity, IR, DM, and viral hepatitis disease. Thus, alterations in PTEN expression and activity in hepatocytes appear to be common and recurrent molecular events associated with liver diseases of various etiologies (Peyrou et al., 2010). PTEN could be a target of emodin (Wu et al., 2022). However, further research is needed to fully understand the emodin-PTEN-inflammation relationship. In the treatment of DM, when HUVECs are chronically exposed to high glucose, oxidative stress occurs within endothelial layer cells, activating apoptotic signaling pathways and contributing to apoptosis and inflammatory responses in endothelial layer cells (Incalza et al., 2018). CCL5 can exert its action by binding to glycosaminoglycans on the surface of endothelial cells, resulting in a powerful chemotactic signal (Suffee et al., 2012). Research indicates that CCL5 is a target of emodin. However, the protective mechanism of p38 MAPK and ERK1/2-based emodin against HUVECs in a high-glucose environment remains to be determined, and it may also be caused by the inhibition of CCL5 (Gao et al., 2015). The study conducted by Lin et al. (2018) showed that CCL5 could promote the activation of p38 MAPK and ERK1/2 (Lin et al., 2018). Therefore, emodin as an inhibitor of CCL5, as a potential drug for the treatment of “vascular disease” caused by DM, is a meaningful study direction. In the treatment of DN, emodin also prevents the development of DN by inhibiting P38 MAPK and NF-κB signaling pathways. Activation of NF-κB and subsequent overexpression of its downstream target transformations TGF-β1 and FN is one of the markers of progressive DN (Yang et al., 2013). P38 MAPK acts as a signal transducer for DN (Tomlinson, 1999; Li et al., 2009). Emodin reduces FN production by inhibiting p38 MAPK phosphorylation and CREB phosphorylation (Li et al., 2009). DNP is the most common complication of DM. Emodin treatment may be involved in the treatment of diabetic neuralgia by mediating P2X3 receptors in the dorsal root ganglia (DRG) (Li L. et al., 2017). Activation of the ERK pathway participates in P2X3 receptor-mediated neuropathic pain (Seino et al., 2006). Emodin may attenuate P2X3 receptor-mediated hyperalgesia by reducing ERK1/2 phosphorylation in the DRG of DM rats (Li L. et al., 2017). In the treatment of OP, emodin inhibits the expression of c-Fos and NFATc1 genes related to osteoclastogenesis by inhibiting receptor activators of NF-κB ligand (RANKL) (Kim et al., 2014). Furthermore, p38 MAPK may be involved in the treatment of OP by emodin. Emodin promotes osteogenesis by regulating the expression of BMP-2 and BMP-9, and it induces BMP-2 gene expression by activating the PI3K-Akt signaling pathway (Lee et al., 2008); the expression of BMP-2 and BMP-9 can induce the phosphorylation of p38 and of mothers against decapentaplegic homolog 1/5/8(SMAD1/5/8), thus stimulating the expression of Runx2 and accelerating the differentiation of osteoblasts (Kim et al., 2014; Daigang et al., 2016; Li X. et al., 2017; Chen et al., 2017).

It is well known that emodin reduces lipid production by activating PPARγ, but interestingly, antagonists of PPARγ have also been shown to inhibit lipogenesis in some studies (Gong et al., 2009; Zhang et al., 2012). Therefore, the regulatory effect of emodin on PPARγ and the lipid-regulating mechanism involved in PPARγ are still worthy of further study.

The targets involved in the treatment of metabolic diseases by emodin deserve attention and further studies can improve the efficiency of clinical research on emodin. miR-20b, FXR, 11β-HSD1 and SREBP can be focused on as key targets of emodin in treating metabolic diseases. Emodin can inhibit the expression of miR-20b. The study conducted by Xiao et al. found that emodin can reduce the overexpression of miR-20b caused by diabetes and IR, thereby improving blood glucose metabolism (Xiao et al., 2019). MicroRNA-20b is also involved in liver lipid metabolism and podocyte apoptosis in DN (Wang X. et al., 2017). Most of the research on emodin has focused on inhibiting FXR targets to treat liver lipid deposition (Shen et al., 2021) and liver inflammation (Iracheta-Vellve et al., 2018). Inhibition of FXR targets can also improve insulin sensitivity, regulate blood glucose (Zhang et al., 2006), and enhance antioxidant capacity (Zhang et al., 2017). Emodin inhibits the 11β-HSD1 target and is mainly used for the treatment of type 2 diabetes. 11β-HSD1 is an attractive therapeutic target for metabolic diseases, with anti-inflammatory properties (Park et al., 2016), lowering blood lipids, increasing glucose tolerance, and enhancing osteoblastogenesis (Feng et al., 2010; Park et al., 2014). SREBP has been proven to play a key role in lipid synthesis (Eberlé et al., 2004). Emodin inhibits the SREBP target and is mainly used for the treatment of obesity, hyperlipidemia, and fatty liver disease (Li J. et al., 2016; He L. F. et al., 2022). In addition, SREBP can also improve type 2 diabetes and IR (Wu and Xiao, 2023). In the study conducted by Fang et al., it was first revealed that emodin inhibits obesity by promoting M2 macrophage polarization through TREM2, and emodin is considered to be a clinical and translational candidate drug for preventing obesity and related metabolic diseases. In recent years, TREM2 has also been (Yu et al., 2021) confirmed to be involved in lipid metabolism (Jaitin et al., 2019) and in high glucose-induced microglial inflammation (Li Y. et al., 2021).

## 6 Study on the pharmacokinetics of emodin

Pharmacokinetics is used to evaluate the safety and effectiveness of drugs, which has guiding significance for the clinical application of emodin. Studies have shown that the pharmacokinetics of oral and injected emodin *in vivo* are consistent with the two-compartment model (Liang et al., 1995; Peng et al., 2008). Pharmacokinetic measurements of emodin are performed by UPLC-MS/MS and HPLC (Tsai and Chen, 1992; Liu et al., 2011). Pharmacokinetics may vary according to sex due to factors such as body weight, plasma volume, gastric emptying time, plasma protein levels, drug transporter function, etc. (Gandhi et al., 2004). The pharmacokinetics of different species will vary due to differences in pH-dependent solubility and first-pass effects (Lin, 1995). The pharmacokinetic parameters of emodin in rabbits and rats of different sexes are shown in Table 4.

**TABLE 4:** Parameters of the pharmacokinetics of emodin.

Route of administration	Species	Dose (mg/kg, equivalent to TSG	Pharmacokinetic parameters								References
			T_max_ (a: h; b: min)	AUC_0-t_ (a: ng.h/ml; b: μg.min/ml)	AUC_0- ∞_(a: ng.h/ml; b: μg.min/ml)	t_1/2_ (h)	V_d_ (L/kg)	C_L_ (a: L/min.kg; b: mL/min.kg)	C_max_ (a: ng/ml; max b: μg/ml)	MRT_0-t_ (h)	
iv	Rabbit (male.SD)	10	N/A	518 ± 355(b)	N/A	3.78	1.03 ± 0.33	72.3 ± 49.2(a)	N/A	N/A	Liang et al. (1995)
oral	Rat (female.SD)	8	18.75 ± 7.51(b)	N/A	33.825 ± 4.09(b)	N/A	N/A	N/A	0.039 ± 0.011(b)	N/A	Liu et al. (2011)
oral	Rat (male.SD)	8	18.00 ± 6.71(b)	N/A	65.70 ± 34.77(b)	N/A	N/A	N/A	0.31 ± 0.094(b)	N/A	
iv	Rat (female.SD)	4	N/A	N/A	282.52 ± 98.42(b)	N/A	N/A	3.98 ± 1.56(b)	N/A	N/A	
iv	Rat (male.SD)	4	N/A	N/A	422.71 ± 163.40(b)	N/A	N/A	2.64 ± 0.86(b)	N/A	N/A	
oral	Rat (male.SD)	34.65	0.75 ± 0.00(a)	624.7 ± 73.35(a)	1108 ± 191.1(a)	23.13 ± 3.56	N/A	N/A	91.65 ± 16.82(a)	28.90 ± 4.71(a)	Li et al. (2019)

Zhou et al. conducted a full-scale pharmacokinetic study of emodin in rats and showed that the absolute bioavailability of emodin was approximately 3.2%, of which 56% was eliminated from the feces as the prototype. The absorbed components were metabolized *in vivo* to hydroxylated and glucuronidated metabolites. The prototypes and metabolites of emodin are mainly distributed in the kidneys. Hydroxylated metabolites are excreted primarily in urine and feces, and glucuronidated metabolites are excreted primarily in urine and bile (Zhou L. et al., 2023). Liang et al. showed that the absolute bioavailability of orally administered emodin was poorer and the rate and extent of absorption were relatively low when compared to intravenous emodin in rabbits, which may be attributed to the very low solubility of emodin in the stomach (Liang et al., 1995). In contrast, Liu et al. attributed the low oral bioavailability of emodin to its extensive distribution of glucuronidation in the intestine and liver (Liu et al., 2012). Despite the low bioavailability of oral emodin, the results of one study indicated that the serum metabolites of intravenous emodin contained emodin glucosinolate, omega-hydroxyhodopsin (omega-OHE), and omega-OHE sulfate/glucosinolate, whereas the serum metabolites of oral emodin were only emodin glucosinolate, although oral emodin had a greater free radical scavenging capacity relative to intravenous emodin (Shia et al., 2010). Thus, emodin glucosinolate may be the active metabolite of emodin. Some studies have indicated that co-administration inhibits the glucuronidation of emodin and enhances the bioavailability of emodin. For example, Di X et al. combined emodin and piperine and found that piperine increased the *in vivo* bioavailability of emodin by inhibiting the glucuronidation of emodin (Di et al., 2015). In addition, co-crystals formed by emodin with other substances can also enhance bioavailability. For example, emodin-nicotinamide co-crystals was two times more soluble than emodin in simulated intestinal fluids, thus enhancing the oral bioavailability of emodin (Ban et al., 2020). In addition, the improved dosage form of emodin increased its bioavailability. The AUC_0-∞_, C_max_, t_1/2_, and MRT_0-∞_of EMO-NE administered orally to rats were 2.37, 1.62, 3.99, and 2.39 times higher relative to emodin suspension, respectively. It was observed that emodin was highest in the liver, followed by lung, kidney, heart and spleen, and lowest in the brain (Shi et al., 2015). The research conducted by Sougiannis AT et al. showed that female mice seemed to metabolize emodin faster than male mice, whether emodin was injected intraperitoneally or orally (Sougiannis et al., 2021). In summary, when conducting clinical research on emodin, comprehensive consideration of sex, dosage form, combination with other substances, and glucuronidation of emodin are the main factors to ensure its effectiveness and safety. Emodin is administered orally and intravenously to treat metabolic diseases. There are few studies on the pharmacological effects of emodin metabolites, and the mechanism of emodin metabolites in treating metabolic diseases *in vivo* is still unclear. The research results of Shia et al. indicated that the metabolites of the two administration methods were different, and there were also differences in the ability of serum metabolites to scavenge free radicals (Shia et al., 2010). Therefore, it is recommended that a systematic study is conducted on the efficacy and mechanism of emodin metabolites with different administration methods in treating metabolic diseases.

## 7 Toxicological analysis

Although emodin has shown promising therapeutic effects in metabolic diseases, in recent years, the effects of emodin have been most widely studied in the liver, kidney, and reproductive system (Dong et al., 2016), which has seriously hindered the development and utilization of emodin.

The hepatotoxicity of emodin has been widely studied and may be related to its metabolism via the liver (Zhou L. et al., 2023). The mechanism of hepatotoxicity of emodin may be related to its severe interference with glutathione (GSH) and fatty acid metabolism in hepatic cells. Emodin-cysteine adducts increase with the concentration of emodin administered, and this combination “depletes” cysteine in the cells and affects GSH synthesis (Liu et al., 2015). On the other hand, emodin induced hepatotoxicity and increased reactive oxidative stress (ROS) levels in mice (emodin:360 mg/kg) and liver cells (emodin:50 μM). **(** Wang et al., 2022 **)**. The hepatotoxicity of emodin is mainly related to the dosage, concentration, and duration of administration. For example, high concentrations of emodin decreased the expression of hepatocyte nuclear factor 4 alpha (HNF4A) and recombinant UDP glucuronosyltransferase 2 family, polypeptide B7 (UGT2B7) in HepG2 cells (emodin: 25, 50 μM) and in rats (emodin:150 mg/kg), which led to hepatotoxicity (Chen et al., 2020). Administered emodin to rats at a dose of 150 mg/kg caused mild inflammation in the liver, but the liver inflammation was further exacerbated by the administration of a dose of 500 mg/kg, and as the dose reached 1,500 mg/kg, liver inflammation was rated as severe and fatty vacuoles began to appear (Yang et al., 2018). Although emodin has shown hepatoprotective effects in many studies, Zheng et al. showed hepatotoxicity when a large concentration of emodin (160 μM) was administered to L02 cells, whereas hepatoprotective effects were observed when emodin concentrations were controlled at 10–80 μM (Zheng et al., 2019). On the other hand, emodin (30 μM) caused apoptosis in L02 cells, and the toxicity increased with longer exposure to emodin (72 h) (Li et al., 2012). It has also been suggested that emodin hepatotoxicity may be related to the coexistence of other substances. For example, the concomitant administration of 1-aminobenzotriazole (ABT) (50 mg/kg) and emodin (80 mg/kg) resulted in severe hepatic injury in mice, which was accompanied by a dramatic increase in hepatic index values, serum gammaglutamyltransferase (GGT) levels, as well as histopathological scores (Zhang et al., 2021). Similarly, Tu C et al. examined the hepatoprotective effects of emodin at doses between 20, 40 and 80 mg/kg in rats, but when a non-toxic concentration of LPS was combined with a safe concentration of emodin, it resulted in a significant increase in plasma ALT and AST, as well as a significant increase in the plasma pro-inflammatory cytokines (TNF-α, IL-1β, and IL-6), and the histology showed liver damage in rats (Tu et al., 2015). Hepatotoxicity of emodin seems to be related to sex in addition to dose, concentration, duration, and co-administration of other drugs. Wu et al. administered emodin (150 mg/kg, 28 days) to rats of different sex and found that the degree of emodin accumulation (AUC) and hepatotoxicity (ALT and AST) were higher in females than in males (Wu et al., 2018).

Hepatotoxicity due to emodin has been reported more often than nephrotoxicity, which may be related to the fact that the liver is the main organ for emodin metabolism (Tarasenko and McGuire, 2017). However, the kidney has been identified as a target organ for emodin toxicity, and emodin has caused renal damage after an increase in the dose of administration. For example, administration of high doses of emodin (0.8 and 1.6 g/kg) caused nephrotoxicity in mice, with nephrotoxicity being more pronounced in the 1.6 g/kg group, but the difference in nephrotoxicity between male and female mice was not significant, and the potential mechanism of toxicity may be related to disruption of oxidative systems, oxidative stress damage, triggering of inflammatory responses, and apoptosis (Huang et al., 2019). In addition, the nephrotoxicity of emodin is also closely related to the time of administration. It took 16 consecutive days of emodin (1,400 mg/kg) administration for rats to show changes in renal pathology, but a lower dose of emodin did not show nephrotoxicity. However, chronic nephrotoxicity was also observed in rats given a low dose of emodin (20 mg/kg) for a prolonged period (14 weeks) (NTP, 2001). It can be seen that the nephrotoxicity of emodin is mainly related to time and dose and not to the sex of the subject. In contrast, a low dose of emodin (10 mg/kg for 9 days) administered for a short period of time was protective against chlorinated paraffin-induced nephrotoxicity in rats (Ali et al., 2013).

Reproductive toxicity of emodin is manifested in various aspects related to reproduction. He et al. incubated zebrafish embryos with emodin at concentrations of 0.25 μg/mL from 7 h to 6 days after fertilization, resulting in a series of phenomena such as edema, trunk curvature, and morphological abnormalities, which were related to emodin metabolites possibly involved in cytochrome P450 3A (CYP3A) and multidrug resistance gene 1 (MDR1) (He et al., 2012). Chang et al. treated mouse blastocyst cells with emodin (25–75 μM, 24 h) and found that emodin appeared to induce damage to mouse blastocysts through an intrinsic apoptotic signaling process, thereby affecting subsequent growth of the embryo (Chang et al., 2012). Several studies have shown that emodin can affect sperm motility in male organisms depending on the dose of emodin. For example, Oshida et al. found that a high dose of emodin (1,000 mg/kg) caused testicular toxicity and affected spermatogenesis and sperm viability in mice (Oshida et al., 2011). Another study indicated that emodin (100, 200, and 400 μM) significantly inhibited total, progressive motility and linear velocity of human spermatozoa. In addition, intracellular Ca^2+^ concentration and tyrosine phosphorylation, which are key regulators of sperm function, decreased in a concentration-dependent manner with emodin (50–400 μM) (Luo et al., 2015). Jahnke et al. administered emodin to rats on gestation days (GD) 6–20 and to mice on GD 6–17 at intake doses of 31, 57, and ∼80–144 mg/kg and 94, 391, and 1,005 mg/kg, respectively. After the termination of pregnancy (rats: GD 20; mice: GD 17), in rats, maternal weight gain during the treatment period was significantly reduced in the high-dose emodin group, but the average weight per fetus was not affected. In mice, the high-dose emodin group showed a decrease in maternal weight and weight gain, and the average weight per fetus was also significantly reduced (Jahnke et al., 2004). The toxicity study of emodin is shown in Table 5.

**TABLE 5:** Study on the toxicity of emodin.

Type of toxicity	Models	Dosage of administration	Duration of administration	Dosing method	References
Hepatotoxicity	Mice	360 mg/kg	30 days	oral	Wang et al. (2022)
	liver cells	50 μM	24 h	indicated	
	Rat	150 mg/kg	28 days	oral	Chen et al. (2020)
	HepG2 cells	25 and 50 μM	48 h	indicated	
	Rat	150, 500, and 1,500 mg/kg	4 weeks	oral	McDonald et al. (2022)
	L02 cells	160 μM	24 h	indicated	Zheng et al. (2019)
Nephrotoxicity	Mice	0.8 and 1.6 g/kg	11 weeks	oral	Huang et al. (2019)
	Rat	20 mg/kg	14 weeks	oral	NTP (2001)
	Rat	1,400 mg/kg	16 days	oral	
Reproductive toxicity	Zebrafish	0.25, 0.5, and 1 μg/ml	72 h	indicated	He et al. (2012)
	Mouse blastocysts	25–75 μM	24 h	indicated	Chang et al. (2012)
	Mice	1,000 mg/kg	5 days	oral	Oshida et al. (2011)
	Human spermatozoa	100, 200, and 400 μM	4 and12 h	indicated	Luo et al. (2015)
	rat	31, 57, and 80–144 mg/kg	14 days	oral	Jahnke et al. (2004)
	mice	94, 391, and 1,005 mg/kg	11 days	oral	

In an experiment using emodin (40, 80, and 160 mg/kg) to treat fatty liver in rats, emodin at a dose of 160 mg/kg showed a strong lipid-lowering effect. However, the liver function test results showed that emodin at doses of 40 mg/kg and 80 mg/kg had a better effect on reducing AST and ALT than 160 mg/kg. It is suggested that the effectiveness of a high dose of emodin on fatty liver cannot avoid the risk of liver function damage. Since the hepatotoxicity of emodin is related to time and dose, and the hepatotoxic dose is close to the therapeutic dose, It is necessary to set multiple administration times and dose gradients to explore the balance between hepatotoxicity and efficacy.

## 8 Discussion and future perspective

Through researching databases including many studies, we found that emodin is primarily involved in metabolic diseases, including obesity, hyperlipidemia, fatty liver, DM and related complications, and OP. This review paper synthesizes the therapeutic effects of emodin on four metabolic diseases and the signaling pathways involved and shows that emodin can participate in metabolic diseases in many ways, including affecting lipid metabolism, enhancing glucose absorption, promoting IR, increasing bone formation ability, and reducing inflammation and autophagy. Emodin involves many signaling pathways, including AMPK, PPAR, and the inflammation signaling pathway. In activating the AMPK signaling pathway, emodin 1) promotes the expression of ACOX1 and CPT-1α genes related to fatty acid oxidation and reduces the expression of SREBP-1 and FAS genes related to lipid synthesis, thereby reducing the expression of liver TG, TC, NEFA, and reducing hepatic lipid deposition; 2) reduces the expression of G6pase, PEPCK, and PGC-1α genes associated with elevated levels of blood glucose, and increases the expression of GLU4 protein, thereby reducing blood glucose levels and enhancing the body’s glucose tolerance; and 3) inhibits autophagy and reduces the apoptosis of glomerulus and renal tubular cells by decreasing the expression of apoptosis-related Bax and Caspase genes. In regulating the PPAR signaling pathway, emodin 1) enhances glucose uptake and lowers blood glucose levels by promoting the expression of GLU4 and GLU1 proteins; 2) inhibits PEPCK and G6PasemRNA expression through regulation of 11β-HSD1, thereby reducing gluconeogenesis; 3) regulates the expression of insulin-sensitivity-related genes, namely, FABPs and resistin, thereby enhancing IR; and 4) promotes the expression of osteogenesis-related genes Runx and osterix, and reduces the expression of lipogenesis-related genes FABPs and C/EBPα, thereby balancing lipid and osteogenesis. The inflammatory signaling pathways inhibited by emodin mainly include the NF-κB signaling pathway and the MAPK signaling pathway, which lead to the following: 1) prevention of fatty liver from progressing to NASH by reducing the release of inflammatory cytokines (IL-1β, IL-6, and TNF-α); 2) improvement in renal function by inhibiting the expression of CREB and FN protein; and 3) inhibition of the expression of osteoclast-related genes C-Fos and NFAC1, and promotion of the expression of osteogenesis-related genes Runx2, thereby inhibiting osteoclast formation and promoting osteoblast differentiation. The regulation of emodin on the AMPK signaling pathway is mainly related to systemic lipid metabolism, the occurrence and development of fatty liver, DM and complications of DN. The target of emodin in the PPAR signaling pathway is mainly concentrated on PPARγ, which is involved in the treatment of DM and OP. In addition, emodin also inhibits a variety of inflammatory signaling pathways and participates in the treatment of fatty liver, DM and its related complications, and OP. Interestingly, the effect of emodin on the regulation of P38 is bidirectional, and the mechanism is currently unclear (Liu et al., 2009; Cui et al., 2016).

Therefore, emodin may serve as a potential drug to regulate lipid and glucose metabolism and promote bone formation. It seems to be more of an energy regulator for the organism. Future research on emodin may involve hyperlipidemia, obesity, fatty liver, DM, atherosclerotic cardiovascular disease, and other fields. The study of the molecular mechanism can be based on the energy regulation of AMPK targets, the glucose metabolism and insulin sensitivity of PPAR targets, and the related targets MAPK and NF-κB involved in the inflammatory signaling pathway. In addition, the study of how emodin affects metabolic diseases can also include the relationship between 11β-HSD1 and lipid-glucose metabolism, and the relationship between PTEN, CCL5, BMP, and inflammatory signaling pathways.

Although the toxicity of emodin has been confirmed by many studies, through a search of the literature we found that the toxicity of emodin is mainly related to the dose of emodin and the time of administration and that the hepatotoxicity is also related to the sex of the subjects. In the treatment of metabolic diseases, emodin is generally administered at doses below 80 and 100 mg/kg in mice and rats, respectively, whereas emodin should cause hepatotoxicity in mice and rats at doses higher than 80 and 150 mg/kg, respectively. The renal injury caused by emodin requires a greater dose and a longer duration of administration. The doses of emodin at which nephrotoxicity occurs in mice and rats should be at least 1,600 and 1,400 mg/kg, respectively. Doses of emodin above 1,000 mg/kg caused testicular toxicity and gestational toxicity in mice, whereas 144 mg/kg of emodin caused little gestational toxicity in mice. Moreover, Lu et al. injected emodin at a dose of 50 mg/kg intraperitoneally into mice for 21 consecutive days, and the results of their hematological toxicity test showed that emodin had almost no effect on the functional indices of the liver, kidneys, and pancreas (Lu et al., 2017), suggesting that it is difficult for emodin to cause biotoxicity at normal doses. Furthermore, in a subchronic toxicity study, administering different doses of emodin (20, 40, and 80 mg/kg) for 12 weeks did not cause pathophysiological disturbances in major organs in mice (Sougiannis et al., 2021). It can be seen that increasing the dose of emodin is the key to the development of biotoxicity compared to the increase in the duration of administration. Therefore, the control of administered doses is particularly important for the development and utilization of emodin.

Although many studies are confirming the use of emodin in the treatment of metabolic diseases, the toxicity of emodin may be the main reason why it is not used as a therapeutic agent in clinical settings. In order to ensure the safety of emodin administration, randomized controlled clinical trials and design-dosing regimens are needed for organisms of different sexes; clinical trials should also consider the safe dose range of emodin administration in different gender groups. According to the results of a pharmacokinetic study of emodin, oral administration of emodin is characterized by low bioavailability, and emodin is unfavorable for absorption and distribution after oral administration because of poor water solubility. Co-crystals can improve the bioavailability of poorly water-soluble drugs, but will not change the pharmacological activity of the drug (McNamara et al., 2006; Emami et al., 2018). It is suggested that co-crystals of emodin should be investigated as antimetabolites to enhance the oral bioavailability of emodin. Another suggestion is that dosage forms of emodin should be improved or its chemical structure should be altered to enhance the solubility of emodin in intestinal fluids, thus enhancing oral bioavailability. There is a lack of studies that comprehensively assess the risk of emodin injections. Although the study conducted by Alexander T. Sougiannis et al. showed no difference in bioavailability between intravenous and oral emodin in mice, the study showed variability in bioavailability depending on the sex of the mice (Sougiannis et al., 2021). Therefore, it is suggested that the examination and comparison of the pharmacological effects and safety of oral and injected emodin metabolites *in vivo* should be taken as the research direction for antimetabolic diseases as well as other pharmacological effects of emodin. Since a large number of molecular targets of emodin increases the complexity of clinical studies on emodin, genomics, transcriptomics, proteomics and metabolomics studies are suggested to screen the differentially expressed genes/proteins/metabolites in order to pinpoint the molecular mechanism of emodin in the treatment of metabolic diseases. In conclusion, the present study provides a comprehensive review and summary of the physicochemical properties, antimetabolic disease effects and mechanisms, pharmacokinetics studies, and toxicity of emodin, in order to provide theoretical evidence for the clinical application of emodin as an antimetabolic disease agent.

## References

[B1] Alberts P. Nilsson C. Selen G. Engblom L. O. Edling N. H. Norling S. (2003). Selective inhibition of 11 beta-hydroxysteroid dehydrogenase type 1 improves hepatic insulin sensitivity in hyperglycemic mice strains. Endocrinology 144 (11), 4755–4762. 10.1210/en.2003-0344 12960099

[B2] Ali B. H. Al-Salam S. Al Husseini I. S. Al-Lawati I. Waly M. Yasin J. (2013). Abrogation of cisplatin-induced nephrotoxicity by emodin in rats. Fundam. Clin. Pharmacol. 27 (2), 192–200. 10.1111/j.1472-8206.2011.01003.x 22044459

[B3] Ban E. An S. H. Park B. Park M. Yoon N. E. Jung B. H. (2020). Improved solubility and oral absorption of emodin-nicotinamide cocrystal over emodin with PVP as a solubility enhancer and crystallization inhibitor. J. Pharm. Sci. 109 (12), 3660–3667. 10.1016/j.xphs.2020.09.030 32987091

[B4] Bessesen D. H. Van Gaal L. F. (2018). Progress and challenges in anti-obesity pharmacotherapy. Lancet Diabetes Endocrinol. 6 (3), 237–248. 10.1016/s2213-8587(17)30236-x 28919062

[B5] Buzzetti E. Pinzani M. Tsochatzis E. A. (2016). The multiple-hit pathogenesis of non-alcoholic fatty liver disease (NAFLD). Metabolism 65 (8), 1038–1048. 10.1016/j.metabol.2015.12.012 26823198

[B6] Byrne C. D. Targher G. (2015). NAFLD: a multisystem disease. J. Hepatol. 62 (1), S47–S64. 10.1016/j.jhep.2014.12.012 25920090

[B7] Cao Y. J. Pu Z. J. Tang Y. P. Shen J. Chen Y. Y. Kang A. (2017). Advances in bio-active constituents, pharmacology and clinical applications of rhubarb. Chin. Med. 12, 36. 10.1186/s13020-017-0158-5 29299052 PMC5745730

[B8] Chang M. H. Huang F. J. Chan W. H. (2012). Emodin induces embryonic toxicity in mouse blastocysts through apoptosis. Toxicology 299 (1), 25–32. 10.1016/j.tox.2012.05.006 22609528

[B9] Chen Z. Zhang L. Yi J. Yang Z. Zhang Z. Li Z. (2012). Promotion of adiponectin multimerization by emodin: a novel AMPK activator with PPARγ-agonist activity. J. Cell. Biochem. 113 (11), 3547–3558. 10.1002/jcb.24232 22730200

[B10] Chen Y. K. Xu Y. K. Zhang H. Yin J. T. Fan X. Liu D. D. (2016). Emodin alleviates jejunum injury in rats with sepsis by inhibiting inflammation response. Biomed. Pharmacother. 84, 1001–1007. 10.1016/j.biopha.2016.10.031 27768925

[B11] Chen X. Zhang S. Chen X. Hu Y. Wu J. Chen S. (2017). Emodin promotes the osteogenesis of MC3T3-E1 cells via BMP-9/Smad pathway and exerts a preventive effect in ovariectomized rats. Acta Biochim. Biophys. Sin. (Shanghai) 49 (10), 867–878. 10.1093/abbs/gmx087 28981600

[B12] Chen Y. Zhang T. Wu L. Huang Y. Mao Z. Zhan Z. (2020). Metabolism and toxicity of emodin: genome-wide association studies reveal hepatocyte nuclear factor 4α regulates UGT2B7 and emodin glucuronidation. Chem. Res. Toxicol. 33 (7), 1798–1808. 10.1021/acs.chemrestox.0c00047 32538071

[B13] Cheng L. Zhang S. Shang F. Ning Y. Huang Z. He R. (2021). Emodin improves glucose and lipid metabolism disorders in obese mice via activating Brown adipose tissue and inducing browning of white adipose tissue. Front. Endocrinol. (Lausanne) 12, 618037. 10.3389/fendo.2021.618037 34040579 PMC8143048

[B14] Cheng D. W. Yue Y. F. Chen C. X. Hu Y. D. Tang Q. Xie M. (2022). Emodin alleviates arthritis pain through reducing spinal inflammation and oxidative stress. Mol. Pain 18, 17448069221146398. 10.1177/17448069221146398 36474308 PMC9772972

[B15] Chinetti-Gbaguidi G. Bouhlel M. A. Copin C. Duhem C. Derudas B. Neve B. (2012). Peroxisome proliferator-activated receptor-γ activation induces 11β-hydroxysteroid dehydrogenase type 1 activity in human alternative macrophages. Arterioscler. Thromb. Vasc. Biol. 32 (3), 677–685. 10.1161/atvbaha.111.241364 22207732 PMC3428270

[B16] Choi S. S. Cha B. Y. Iida K. Lee Y. S. Yonezawa T. Teruya T. (2011). Artepillin C, as a PPARγ ligand, enhances adipocyte differentiation and glucose uptake in 3T3-L1 cells. Biochem. Pharmacol. 81 (7), 925–933. 10.1016/j.bcp.2011.01.002 21219874

[B17] Cui Y. Lu P. Song G. Liu Q. Zhu D. Liu X. (2016). Involvement of PI3K/Akt, ERK and p38 signaling pathways in emodin-mediated extrinsic and intrinsic human hepatoblastoma cell apoptosis. Food Chem. Toxicol. 92, 26–37. 10.1016/j.fct.2016.03.013 27032576

[B18] Cui Y. R. Bu Z. Q. Yu H. Y. Yan L. L. Feng J. (2023). Emodin attenuates inflammation and demyelination in experimental autoimmune encephalomyelitis. Neural Regen. Res. 18 (7), 1535–1541. 10.4103/1673-5374.358612 36571359 PMC10075100

[B19] Daigang L. Jining Q. Jinlai L. Pengfei W. Chuan S. Liangku H. (2016). LPS-stimulated inflammation inhibits BMP-9-induced osteoblastic differentiation through crosstalk between BMP/MAPK and Smad signaling. Exp. Cell. Res. 341 (1), 54–60. 10.1016/j.yexcr.2016.01.009 26794904

[B20] Day E. A. Ford R. J. Steinberg G. R. (2017). AMPK as a therapeutic target for treating metabolic diseases. Trends Endocrinol. Metab. 28 (8), 545–560. 10.1016/j.tem.2017.05.004 28647324

[B21] de Sereday M. S. Gonzalez C. Giorgini D. De Loredo L. Braguinsky J. Cobeñas C. (2004). Prevalence of diabetes, obesity, hypertension and hyperlipidemia in the central area of Argentina. Diabetes Metab. 30 (4), 335–339. 10.1016/s1262-3636(07)70125-8 15525876

[B22] Di X. Wang X. Di X. Liu Y. (2015). Effect of piperine on the bioavailability and pharmacokinetics of emodin in rats. J. Pharm. Biomed. Anal. 115, 144–149. 10.1016/j.jpba.2015.06.027 26201645

[B23] Dong H. Lu F. E. Gao Z. Q. Xu L. J. Wang K. F. Zou X. (2005). Effects of emodin on treating murine nonalcoholic fatty liver induced by high caloric laboratory chaw. World J. Gastroenterol. 11 (9), 1339–1344. 10.3748/wjg.v11.i9.1339 15761972 PMC4250681

[B24] Dong X. Fu J. Yin X. Cao S. Li X. Lin L. (2016). Emodin: a review of its pharmacology, toxicity and pharmacokinetics. Phytother. Res. 30 (8), 1207–1218. 10.1002/ptr.5631 27188216 PMC7168079

[B25] Duarte N. Coelho I. C. Patarrão R. S. Almeida J. I. Penha-Gonçalves C. Macedo M. P. (2015). How inflammation impinges on NAFLD: a role for kupffer cells. Biomed. Res. Int. 2015, 984578. 10.1155/2015/984578 26090470 PMC4450298

[B26] Ducy P. Starbuck M. Priemel M. Shen J. Pinero G. Geoffroy V. (1999). A Cbfa1-dependent genetic pathway controls bone formation beyond embryonic development. Genes. Dev. 13 (8), 1025–1036. 10.1101/gad.13.8.1025 10215629 PMC316641

[B27] Đukanović S. Ganić T. Lončarević B. Cvetković S. Nikolić B. Tenji D. (2022). Elucidating the antibiofilm activity of Frangula emodin against *Staphylococcus aureus* biofilms. J. Appl. Microbiol. 132 (3), 1840–1855. 10.1111/jam.15360 34779074

[B28] Eberlé D. Hegarty B. Bossard P. Ferré P. Foufelle F. (2004). SREBP transcription factors: master regulators of lipid homeostasis. Biochimie 86 (11), 839–848. 10.1016/j.biochi.2004.09.018 15589694

[B29] El-Jack A. K. Hamm J. K. Pilch P. F. Farmer S. R. (1999). Reconstitution of insulin-sensitive glucose transport in fibroblasts requires expression of both PPARgamma and C/EBPalpha. J. Biol. Chem. 274 (12), 7946–7951. 10.1074/jbc.274.12.7946 10075691

[B30] Emami S. Siahi-Shadbad M. Adibkia K. Barzegar-Jalali M. (2018). Recent advances in improving oral drug bioavailability by cocrystals. Bioimpacts 8 (4), 305–320. 10.15171/bi.2018.33 30397585 PMC6209825

[B31] Feng Y. Huang S. L. Dou W. Zhang S. Chen J. H. Shen Y. (2010). Emodin, a natural product, selectively inhibits 11beta-hydroxysteroid dehydrogenase type 1 and ameliorates metabolic disorder in diet-induced obese mice. Br. J. Pharmacol. 161 (1), 113–126. 10.1111/j.1476-5381.2010.00826.x 20718744 PMC2962821

[B32] Forbes J. M. Cooper M. E. (2013). Mechanisms of diabetic complications. Physiol. Rev. 93 (1), 137–188. 10.1152/physrev.00045.2011 23303908

[B33] Friedman S. L. Neuschwander-Tetri B. A. Rinella M. Sanyal A. J. (2018). Mechanisms of NAFLD development and therapeutic strategies. Nat. Med. 24 (7), 908–922. 10.1038/s41591-018-0104-9 29967350 PMC6553468

[B34] Fruchart J. C. Staels B. Duriez P. (2001). PPARS, metabolic disease and atherosclerosis. Pharmacol. Res. 44 (5), 345–352. 10.1006/phrs.2001.0871 11712864

[B35] Fu X. Xu A. G. Yao M. Y. Guo L. Zhao L. S. (2014). Emodin enhances cholesterol efflux by activating peroxisome proliferator-activated receptor-γ in oxidized low density lipoprotein-loaded THP1 macrophages. Clin. Exp. Pharmacol. Physiol. 41 (9), 679–684. 10.1111/1440-1681.12262 24837536

[B36] Gandhi M. Aweeka F. Greenblatt R. M. Blaschke T. F. (2004). Sex differences in pharmacokinetics and pharmacodynamics. Annu. Rev. Pharmacol. Toxicol. 44, 499–523. 10.1146/annurev.pharmtox.44.101802.121453 14744256

[B37] Gao Y. Zhang J. Li G. Xu H. Yi Y. Wu Q. (2015). Protection of vascular endothelial cells from high glucose-induced cytotoxicity by emodin. Biochem. Pharmacol. 94 (1), 39–45. 10.1016/j.bcp.2015.01.006 25619422

[B38] Gao R. Wang C. Han A. Tian Y. Ren S. Lv W. (2021). Emodin improves intestinal health and immunity through modulation of gut microbiota in mice infected by pathogenic *Escherichia coli* O(1). Anim. (Basel) 11 (11). 10.3390/ani11113314 PMC8614316 34828045

[B39] Gong Z. Huang C. Sheng X. Zhang Y. Li Q. Wang M. W. (2009). The role of tanshinone IIA in the treatment of obesity through peroxisome proliferator-activated receptor gamma antagonism. Endocrinology 150 (1), 104–113. 10.1210/en.2008-0322 18818299

[B40] Guo X. Liu J. Cai S. Wang O. Ji B. (2016). Synergistic interactions of apigenin, naringin, quercetin and emodin on inhibition of 3T3-L1 preadipocyte differentiation and pancreas lipase activity. Obes. Res. Clin. Pract. 10 (3), 327–339. 10.1016/j.orcp.2015.08.004 26314502

[B41] He Q. Liu K. Wang S. Hou H. Yuan Y. Wang X. (2012). Toxicity induced by emodin on zebrafish embryos. Drug Chem. Toxicol. 35 (2), 149–154. 10.3109/01480545.2011.589447 21834668

[B42] He L. Yang F. Q. Tang P. Gao T. H. Yang C. X. Tan L. (2022a). Regulation of the intestinal flora: a potential mechanism of natural medicines in the treatment of type 2 diabetes mellitus. Biomed. Pharmacother. 151, 113091. 10.1016/j.biopha.2022.113091 35576662

[B43] He L. F. Wang C. Zhang Y. F. Guo C. C. Wan Y. Li Y. X. (2022b). Effect of emodin on hyperlipidemia and hepatic lipid metabolism in zebrafish larvae fed a high-cholesterol diet. Chem. Biodivers. 19 (2), e202100675. 10.1002/cbdv.202100675 34866324

[B44] Herzig S. Shaw R. J. (2018). AMPK: guardian of metabolism and mitochondrial homeostasis. Nat. Rev. Mol. Cell. Biol. 19 (2), 121–135. 10.1038/nrm.2017.95 28974774 PMC5780224

[B45] Hsu S. C. Chung J. G. (2012). Anticancer potential of emodin. Biomed. (Taipei) 2 (3), 108–116. 10.1016/j.biomed.2012.03.003 PMC7104001 32289000

[B46] Hu N. Liu J. Xue X. Li Y. (2020). The effect of emodin on liver disease -- comprehensive advances in molecular mechanisms. Eur. J. Pharmacol. 882, 173269. 10.1016/j.ejphar.2020.173269 32553811

[B47] Hu Q. Yao J. Wu X. Li J. Li G. Tang W. (2022). Emodin attenuates severe acute pancreatitis-associated acute lung injury by suppressing pancreatic exosome-mediated alveolar macrophage activation. Acta Pharm. Sin. B 12 (10), 3986–4003. 10.1016/j.apsb.2021.10.008 36213542 PMC9532455

[B48] Hua S. Liu F. Wang M. (2019). Emodin alleviates the airway inflammation of cough variant asthma in mice by regulating the Notch pathway. Med. Sci. Monit. 25, 5621–5629. 10.12659/msm.915080 31354164 PMC6685324

[B49] Huang W. Li Y. Jiang Q. Luo Y. Ai X. Wang P. (2019). Apoptosis mechanism of emodin on renal toxicity in mice. Chin. J. Exp. Traditional Med. Formulae, 42–47. 10.13422/j.cnki.syfjx.20182421

[B50] Iglesias P. Díez J. J. (2006). Peroxisome proliferator-activated receptor gamma agonists in renal disease. Eur. J. Endocrinol. 154 (5), 613–621. 10.1530/eje.1.02134 16645006

[B51] Incalza M. A. D'Oria R. Natalicchio A. Perrini S. Laviola L. Giorgino F. (2018). Oxidative stress and reactive oxygen species in endothelial dysfunction associated with cardiovascular and metabolic diseases. Vasc. Pharmacol. 100, 1–19. 10.1016/j.vph.2017.05.005 28579545

[B52] Iracheta-Vellve A. Calenda C. D. Petrasek J. Ambade A. Kodys K. Adorini L. (2018). FXR and TGR5 agonists ameliorate liver injury, steatosis, and inflammation after binge or prolonged alcohol feeding in mice. Hepatol. Commun. 2 (11), 1379–1391. 10.1002/hep4.1256 30411084 PMC6211332

[B53] Jahnke G. D. Price C. J. Marr M. C. Myers C. B. George J. D. (2004). Developmental toxicity evaluation of emodin in rats and mice. Birth Defects Res. B Dev. Reprod. Toxicol. 71 (2), 89–101. 10.1002/bdrb.20002 15098202

[B54] Jaitin D. A. Adlung L. Thaiss C. A. Weiner A. Li B. Descamps H. (2019). Lipid-associated macrophages control metabolic homeostasis in a trem2-dependent manner. Cell. 178 (3), 686–698.e614. 10.1016/j.cell.2019.05.054 31257031 PMC7068689

[B55] Janke J. Engeli S. Gorzelniak K. Luft F. C. Sharma A. M. (2002). Resistin gene expression in human adipocytes is not related to insulin resistance. Obes. Res. 10 (1), 1–5. 10.1038/oby.2002.1 11786595

[B56] Ji J. Tao P. Wang Q. Cui M. Cao M. Xu Y. (2023). Emodin attenuates diabetic kidney disease by inhibiting ferroptosis via upregulating Nrf2 expression. Aging (Albany NY) 15 (15), 7673–7688. 10.18632/aging.204933 37552124 PMC10457067

[B57] Jia X. Iwanowycz S. Wang J. Saaoud F. Yu F. Wang Y. (2014). Emodin attenuates systemic and liver inflammation in hyperlipidemic mice administrated with lipopolysaccharides. Exp. Biol. Med. (Maywood) 239 (8), 1025–1035. 10.1177/1535370214530247 24740873 PMC4988953

[B58] Jin T. Ai F. Zhou J. Kong L. Xiong Z. Wang D. (2023). Emodin alleviates lung ischemia-reperfusion injury by suppressing gasdermin D-mediated pyroptosis in rats. Clin. Respir. J. 17 (3), 241–250. 10.1111/crj.13582 36751097 PMC9978909

[B59] Kang D. M. Yoon K. H. Kim J. Y. Oh J. M. Lee M. Jung S. T. (2014). CT imaging biomarker for evaluation of emodin as a potential drug on LPS-mediated osteoporosis mice. Acad. Radiol. 21 (4), 457–462. 10.1016/j.acra.2013.12.009 24594415

[B60] Kanis J. A. Melton L. J. 3rd Christiansen C. Johnston C. C. Khaltaev N. (1994). The diagnosis of osteoporosis. J. Bone Min. Res. 9 (8), 1137–1141. 10.1002/jbmr.5650090802 7976495

[B61] Kim J. Y. Cheon Y. H. Kwak S. C. Baek J. M. Yoon K. H. Lee M. S. (2014). Emodin regulates bone remodeling by inhibiting osteoclastogenesis and stimulating osteoblast formation. J. Bone Min. Res. 29 (7), 1541–1553. 10.1002/jbmr.2183 25832436

[B62] King G. L. (2008). The role of inflammatory cytokines in diabetes and its complications. J. Periodontol. 79 (8), 1527–1534. 10.1902/jop.2008.080246 18673007

[B63] Komori T. (2003). Requisite roles of Runx2 and Cbfb in skeletal development. J. Bone Min. Metab. 21 (4), 193–197. 10.1007/s00774-002-0408-0 12811622

[B64] Kuryłowicz A. Puzianowska-Kuźnicka M. (2020). Induction of adipose tissue browning as a strategy to combat obesity. Int. J. Mol. Sci. 21 (17). 10.3390/ijms21176241 PMC7504355 32872317

[B65] Lau L. H. S. Wong S. H. (2018). Microbiota, obesity and NAFLD. Adv. Exp. Med. Biol. 1061, 111–125. 10.1007/978-981-10-8684-7_9 29956210

[B66] Lee J. H. Chan J. L. Yiannakouris N. Kontogianni M. Estrada E. Seip R. (2003). Circulating resistin levels are not associated with obesity or insulin resistance in humans and are not regulated by fasting or leptin administration: cross-sectional and interventional studies in normal, insulin-resistant, and diabetic subjects. J. Clin. Endocrinol. Metab. 88 (10), 4848–4856. 10.1210/jc.2003-030519 14557464

[B67] Lee S. U. Shin H. K. Min Y. K. Kim S. H. (2008). Emodin accelerates osteoblast differentiation through phosphatidylinositol 3-kinase activation and bone morphogenetic protein-2 gene expression. Int. Immunopharmacol. 8 (5), 741–747. 10.1016/j.intimp.2008.01.027 18387517

[B68] Lee J. W. Park S. Takahashi Y. Wang H. G. (2010). The association of AMPK with ULK1 regulates autophagy. PLoS One 5 (11), e15394. 10.1371/journal.pone.0015394 21072212 PMC2972217

[B69] Lee E. H. Baek S. Y. Park J. Y. Kim Y. W. (2020). Emodin in Rheum undulatum inhibits oxidative stress in the liver via AMPK with Hippo/Yap signalling pathway. Pharm. Biol. 58 (1), 333–341. 10.1080/13880209.2020.1750658 32306810 PMC7191907

[B70] Li X. Liu W. Wang Q. Liu P. Deng Y. Lan T. (2009). Emodin suppresses cell proliferation and fibronectin expression via p38MAPK pathway in rat mesangial cells cultured under high glucose. Mol. Cell. Endocrinol. 307 (1-2), 157–162. 10.1016/j.mce.2009.03.006 19524136

[B71] Li C. L. Ma J. Zheng L. Li H. J. Li P. (2012). Determination of emodin in L-02 cells and cell culture media with liquid chromatography-mass spectrometry: application to a cellular toxicokinetic study. J. Pharm. Biomed. Anal. 71, 71–78. 10.1016/j.jpba.2012.07.031 22944356

[B72] Li X. Xu M. Wang F. Ji Y. Davidso N. W. Li Z. (2015a). Interaction of ApoA-IV with NR4A1 and NR1D1 represses G6Pase and PEPCK transcription: nuclear receptor-mediated downregulation of hepatic gluconeogenesis in mice and a human hepatocyte cell line. PLoS One 10 (11), e0142098. 10.1371/journal.pone.0142098 26556724 PMC4640595

[B73] Li Y. Xiong W. Yang J. Zhong J. Zhang L. Zheng J. (2015b). Attenuation of inflammation by emodin in lipopolysaccharide-induced acute kidney injury via inhibition of toll-like receptor 2 signal pathway. Iran. J. Kidney Dis. 9 (3), 202–208. http://www.ijkd.org/index.php/ijkd/article/view/1676/771 . 25957424

[B74] Li J. Ding L. Song B. Xiao X. Qi M. Yang Q. (2016a). Emodin improves lipid and glucose metabolism in high fat diet-induced obese mice through regulating SREBP pathway. Eur. J. Pharmacol. 770, 99–109. 10.1016/j.ejphar.2015.11.045 26626587

[B75] Li X. Xu Z. Wang S. Guo H. Dong S. Wang T. (2016b). Emodin ameliorates hepatic steatosis through endoplasmic reticulum-stress sterol regulatory element-binding protein 1c pathway in liquid fructose-feeding rats. Hepatol. Res. 46 (3), E105–E117. 10.1111/hepr.12538 26031413

[B76] Li L. Sheng X. Zhao S. Zou L. Han X. Gong Y. (2017a). Nanoparticle-encapsulated emodin decreases diabetic neuropathic pain probably via a mechanism involving P2X3 receptor in the dorsal root ganglia. Purinergic Signal 13 (4), 559–568. 10.1007/s11302-017-9583-2 28840511 PMC5714846

[B77] Li X. Guo L. Liu Y. Su Y. Xie Y. Du J. (2017b). MicroRNA-21 promotes osteogenesis of bone marrow mesenchymal stem cells via the Smad7-Smad1/5/8-Runx2 pathway. Biochem. Biophys. Res. Commun. 493 (2), 928–933. 10.1016/j.bbrc.2017.09.119 28943430

[B78] Li R. R. Liu X. F. Feng S. X. Shu S. N. Wang P. Y. Zhang N. (2019). Pharmacodynamics of five anthraquinones (Aloe-emodin, emodin, rhein, chysophanol, and physcion) and reciprocal pharmacokinetic interaction in rats with cerebral ischemia. Molecules 24 (10). 10.3390/molecules24101898 PMC6571683 31108858

[B79] Li R. Liu W. Ou L. Gao F. Li M. Wang L. (2020a). Emodin alleviates hydrogen peroxide-induced inflammation and oxidative stress via mitochondrial dysfunction by inhibiting the PI3K/mTOR/GSK3β pathway in neuroblastoma SH-SY5Y cells. Biomed. Res. Int. 2020, 1562915. 10.1155/2020/1562915 32832542 PMC7428951

[B80] Li X. Shan C. Wu Z. Yu H. Yang A. Tan B. (2020b). Emodin alleviated pulmonary inflammation in rats with LPS-induced acute lung injury through inhibiting the mTOR/HIF-1α/VEGF signaling pathway. Inflamm. Res. 69 (4), 365–373. 10.1007/s00011-020-01331-3 32130427

[B81] Li W. Wang D. Li M. Li B. (2021a). Emodin inhibits the proliferation of papillary thyroid carcinoma by activating AMPK. Exp. Ther. Med. 22 (4), 1075. 10.3892/etm.2021.10509 34447468 PMC8355685

[B82] Li Y. Long W. Gao M. Jiao F. Chen Z. Liu M. (2021b). TREM2 regulates high glucose-induced microglial inflammation via the NLRP3 signaling pathway. Brain Sci. 11 (7). 10.3390/brainsci11070896 PMC8306970 34356130

[B83] Li F. Song X. Zhou X. Chen L. Zheng J. (2023). Emodin attenuates high lipid-induced liver metastasis through the AKT and ERK pathways *in vitro* in breast cancer cells and in a mouse xenograft model. Heliyon 9 (6), e17052. 10.1016/j.heliyon.2023.e17052 37484373 PMC10361095

[B84] Liang J. Hsiu S. Wu P. Chao P. (1995). Emodin pharmacokinetics in rabbits. Planta medica. 61 (05), 406–408. 10.1055/s-2006-958125 7480199

[B85] Lin L. Ni B. Lin H. Zhang M. Li X. Yin X. (2015). Traditional usages, botany, phytochemistry, pharmacology and toxicology of Polygonum multiflorum Thunb. a review. J. Ethnopharmacol. 159, 158–183. 10.1016/j.jep.2014.11.009 25449462 PMC7127521

[B86] Lin C. S. Hsieh P. S. Hwang L. L. Lee Y. H. Tsai S. H. Tu Y. C. (2018). The CCL5/CCR5 Axis promotes vascular smooth muscle cell proliferation and atherogenic phenotype switching. Cell. Physiol. Biochem. 47 (2), 707–720. 10.1159/000490024 29794461

[B87] Lin J. H. (1995). Species similarities and differences in pharmacokinetics. Drug Metab. Dispos. 23 (10), 1008–1021. https://dmd.aspetjournals.org/content/23/10/1008.long . 8654187

[B88] Liu Y. Jia L. Liu Z. C. Zhang H. Zhang P. J. Wan Q. (2009). Emodin ameliorates high-glucose induced mesangial p38 over-activation and hypocontractility via activation of PPARgamma. Exp. Mol. Med. 41 (9), 648–655. 10.3858/emm.2009.41.9.071 19478555 PMC2753658

[B89] Liu W. Zheng Z. Liu X. Gao S. Ye L. Yang Z. (2011). Sensitive and robust UPLC-MS/MS method to determine the gender-dependent pharmacokinetics in rats of emodin and its glucuronide. J. Pharm. Biomed. Anal. 54 (5), 1157–1162. 10.1016/j.jpba.2010.12.004 21195574 PMC4010304

[B90] Liu W. Feng Q. Li Y. Ye L. Hu M. Liu Z. (2012). Coupling of UDP-glucuronosyltransferases and multidrug resistance-associated proteins is responsible for the intestinal disposition and poor bioavailability of emodin. Toxicol. Appl. Pharmacol. 265 (3), 316–324. 10.1016/j.taap.2012.08.032 22982073

[B91] Liu X. Liu Y. Qu Y. Cheng M. Xiao H. (2015). Metabolomic profiling of emodin-induced cytotoxicity in human liver cells and mechanistic study. Toxicol. Res. 4. 10.1039/C4TX00246F

[B92] Liu H. Wang Q. Shi G. Yang W. Zhang Y. Chen W. (2021). Emodin ameliorates renal damage and podocyte injury in a rat model of diabetic nephropathy via regulating AMPK/mTOR-Mediated autophagy signaling pathway. Diabetes Metab. Syndr. Obes. 14, 1253–1266. 10.2147/dmso.S299375 33776462 PMC7987270

[B93] Liu J. Wu A. Cai J. She Z. G. Li H. (2022a). The contribution of the gut-liver axis to the immune signaling pathway of NAFLD. Front. Immunol. 13, 968799. 10.3389/fimmu.2022.968799 36119048 PMC9471422

[B94] Liu Y. Li M. Teh L. Zhao L. Ye N. Wu L. (2022b). Emodin-mediated treatment of acute kidney injury. Evid. Based Complement. Altern. Med. 2022, 5699615. 10.1155/2022/5699615 PMC8959961 35356249

[B95] Long H. Chen H. Yan J. Cheng H. (2022). Emodin exerts antitumor effects in ovarian cancer cell lines by preventing the development of cancer stem cells via epithelial mesenchymal transition. Oncol. Lett. 23 (3), 95. 10.3892/ol.2022.13215 35154426 PMC8822392

[B96] Lu Y. Jeong Y. T. Li X. Kim M. J. Park P. H. Hwang S. L. (2013). Emodin isolated from polygoni cuspidati radix inhibits TNF-α and IL-6 release by blockading NF-κB and MAP kinase pathways in mast cells stimulated with PMA plus A23187. Biomol. Ther. Seoul. 21 (6), 435–441. 10.4062/biomolther.2013.068 24404333 PMC3879914

[B97] Lu K. Xie S. Han S. Zhang J. Chang X. Chao J. (2014). Preparation of a nano emodin transfersome and study on its anti-obesity mechanism in adipose tissue of diet-induced obese rats. J. Transl. Med. 12, 72. 10.1186/1479-5876-12-72 24641917 PMC3994574

[B98] Lu J. Xu Y. Zhao Z. Ke X. Wei X. Kang J. (2017). Emodin suppresses proliferation, migration and invasion in ovarian cancer cells by down regulating ILK *in vitro* and *in vivo* . Onco Targets Ther. 10, 3579–3589. 10.2147/ott.S138217 28790850 PMC5530856

[B99] Luo T. Li N. He Y. Q. Weng S. Q. Wang T. Zou Q. X. (2015). Emodin inhibits human sperm functions by reducing sperm [Ca(2+)]i and tyrosine phosphorylation. Reprod. Toxicol. 51, 14–21. 10.1016/j.reprotox.2014.11.007 25463531

[B100] Luo J. S. Zhao X. Yang Y. (2020). Effects of emodin on inflammatory bowel disease-related osteoporosis. Biosci. Rep. 40 (1). 10.1042/bsr20192317 PMC6992925 31934719

[B101] Luo S. He J. Huang S. Wang X. Su Y. Li Y. (2022). Emodin targeting the colonic metabolism via PPARγ alleviates UC by inhibiting facultative anaerobe. Phytomedicine 104, 154106. 10.1016/j.phymed.2022.154106 35728384

[B102] Mabwi H. A. Lee H. J. Hitayezu E. Mauliasari I. R. Pan C. H. Mwaikono K. S. (2023). Emodin modulates gut microbial community and triggers intestinal immunity. J. Sci. Food Agric. 103 (3), 1273–1282. 10.1002/jsfa.12221 36088620 PMC10087506

[B103] Martorell M. Castro N. Victoriano M. Capó X. Tejada S. Vitalini S. (2021). An update of anthraquinone derivatives emodin, diacerein, and catenarin in diabetes. Evid. Based Complement. Altern. Med. 2021, 3313419. 10.1155/2021/3313419 PMC8476274 34589130

[B104] McDonald S. J. VanderVeen B. N. Velazquez K. T. Enos R. T. Fairman C. M. Cardaci T. D. (2022). Therapeutic potential of emodin for gastrointestinal cancers. Integr. Cancer Ther. 21, 15347354211067469. 10.1177/15347354211067469 34984952 PMC8738880

[B105] McGee S. L. van Denderen B. J. Howlett K. F. Mollica J. Schertzer J. D. Kemp B. E. (2008). AMP-activated protein kinase regulates GLUT4 transcription by phosphorylating histone deacetylase 5. Diabetes 57 (4), 860–867. 10.2337/db07-0843 18184930

[B106] McNamara D. P. Childs S. L. Giordano J. Iarriccio A. Cassidy J. Shet M. S. (2006). Use of a glutaric acid cocrystal to improve oral bioavailability of a low solubility API. Pharm. Res. 23 (8), 1888–1897. 10.1007/s11095-006-9032-3 16832611

[B107] Mihaylova M. M. Shaw R. J. (2011). The AMPK signalling pathway coordinates cell growth, autophagy and metabolism. Nat. Cell. Biol. 13 (9), 1016–1023. 10.1038/ncb2329 21892142 PMC3249400

[B108] Montaigne D. Butruille L. Staels B. (2021). PPAR control of metabolism and cardiovascular functions. Nat. Rev. Cardiol. 18 (12), 809–823. 10.1038/s41569-021-00569-6 34127848

[B109] Moschen A. R. Kaser A. Enrich B. Ludwiczek O. Gabriel M. Obrist P. (2005). The RANKL/OPG system is activated in inflammatory bowel disease and relates to the state of bone loss. Gut 54 (4), 479–487. 10.1136/gut.2004.044370 15753532 PMC1774465

[B110] Muir L. A. Neeley C. K. Meyer K. A. Baker N. A. Brosius A. M. Washabaugh A. R. (2016). Adipose tissue fibrosis, hypertrophy, and hyperplasia: correlations with diabetes in human obesity. Obes. (Silver Spring) 24 (3), 597–605. 10.1002/oby.21377 PMC4920141 26916240

[B111] Mundy G. R. (2007). Osteoporosis and inflammation. Nutr. Rev. 65 (12), S147–S151. 10.1111/j.1753-4887.2007.tb00353.x 18240539

[B112] Nakashima K. Zhou X. Kunkel G. Zhang Z. Deng J. M. Behringer R. R. (2002). The novel zinc finger-containing transcription factor osterix is required for osteoblast differentiation and bone formation. Cell. 108 (1), 17–29. 10.1016/s0092-8674(01)00622-5 11792318

[B113] NTP (2001). NTP toxicology and carcinogenesis studies of EMODIN (CAS NO. 518-82-1) feed studies in F344/N rats and B6C3F1 mice. Natl. Toxicol. Program Tech. Rep. Ser. 493, 1–278. https://ntp.niehs.nih.gov/go/tr493abs . 12563347

[B114] Oshida K. Hirakata M. Maeda A. Miyoshi T. Miyamoto Y. (2011). Toxicological effect of emodin in mouse testicular gene expression profile. J. Appl. Toxicol. 31 (8), 790–800. 10.1002/jat.1637 21319176

[B115] Ovens A. J. Scott J. W. Langendorf C. G. Kemp B. E. Oakhill J. S. Smiles W. J. (2021). Post-translational modifications of the energy guardian AMP-activated protein kinase. Int. J. Mol. Sci. 22 (3). 10.3390/ijms22031229 PMC7866021 33513781

[B116] Pang X. Liu J. Li Y. Zhao J. Zhang X. (2015). Emodin inhibits homocysteine-induced C-reactive protein generation in vascular smooth muscle cells by regulating PPARγ expression and ROS-ERK1/2/p38 signal pathway. PLoS One 10 (7), e0131295. 10.1371/journal.pone.0131295 26131983 PMC4488440

[B117] Park J. S. Bae S. J. Choi S. W. Son Y. H. Park S. B. Rhee S. D. (2014). A novel 11β-HSD1 inhibitor improves diabesity and osteoblast differentiation. J. Mol. Endocrinol. 52 (2), 191–202. 10.1530/jme-13-0177 24444497

[B118] Park S. B. Park J. S. Jung W. H. Kim H. Y. Kwak H. J. Ahn J. H. (2016). Anti-inflammatory effect of a selective 11β-hydroxysteroid dehydrogenase type 1 inhibitor via the stimulation of heme oxygenase-1 in LPS-activated mice and J774.1 murine macrophages. J. Pharmacol. Sci. 131 (4), 241–250. 10.1016/j.jphs.2016.07.003 27523796

[B119] Park S. Y. Choi Y. W. Park G. (2018). Nrf2-mediated neuroprotection against oxygen-glucose deprivation/reperfusion injury by emodin via AMPK-dependent inhibition of GSK-3β. J. Pharm. Pharmacol. 70 (4), 525–535. 10.1111/jphp.12885 29424025

[B120] Pelton P. D. Zhou L. Demarest K. T. Burris T. P. (1999). PPARgamma activation induces the expression of the adipocyte fatty acid binding protein gene in human monocytes. Biochem. Biophys. Res. Commun. 261 (2), 456–458. 10.1006/bbrc.1999.1071 10425206

[B121] Peng j. Song Z. Ma C. (2008). Emodin Studies on Pharmacokinetics and Distribution in Rat Liver after Polygonum cuspidatum Sieb. et Zucc. Extract Administration. World Sci. Technol. 10 (1), 64–67. 10.1016/S1876-3553(09)60004-1

[B122] Peng S. Wang J. Lu C. Xu Z. Chai J. J. Ke Q. (2021). Emodin enhances cisplatin sensitivity in non-small cell lung cancer through Pgp downregulation. Oncol. Lett. 21 (3), 230. 10.3892/ol.2021.12491 33613719 PMC7856686

[B123] Peyrou M. Bourgoin L. Foti M. (2010). PTEN in non-alcoholic fatty liver disease/non-alcoholic steatohepatitis and cancer. Dig. Dis. 28 (1), 236–246. 10.1159/000282095 20460918

[B124] Pietropaolo M. Le Roith D. (2001). Pathogenesis of diabetes: our current understanding. Clin. Cornerstone 4 (2), 1–16. 10.1016/s1098-3597(01)90025-0 11838323

[B125] Qin B. Zeng Z. Xu J. Shangwen J. Ye Z. J. Wang S. (2022). Emodin inhibits invasion and migration of hepatocellular carcinoma cells via regulating autophagy-mediated degradation of snail and β-catenin. BMC Cancer 22 (1), 671. 10.1186/s12885-022-09684-0 35715752 PMC9206273

[B126] Rochette L. Zeller M. Cottin Y. Vergely C. (2014). Diabetes, oxidative stress and therapeutic strategies. Biochim. Biophys. Acta 1840 (9), 2709–2729. 10.1016/j.bbagen.2014.05.017 24905298

[B127] Saunders I. T. Mir H. Kapur N. Singh S. (2019). Emodin inhibits colon cancer by altering BCL-2 family proteins and cell survival pathways. Cancer Cell. Int. 19, 98. 10.1186/s12935-019-0820-3 31011292 PMC6466701

[B128] Schulte C. M. Dignass A. U. Goebell H. Röher H. D. Schulte K. M. (2000). Genetic factors determine extent of bone loss in inflammatory bowel disease. Gastroenterology 119 (4), 909–920. 10.1053/gast.2000.18158 11040178

[B129] Seeman E. (2003). Invited review: pathogenesis of osteoporosis. J. Appl. Physiol. (1985) 95(5), 2142–2151. 10.1152/japplphysiol.00564.2003 14555675

[B130] Seino D. Tokunaga A. Tachibana T. Yoshiya S. Dai Y. Obata K. (2006). The role of ERK signaling and the P2X receptor on mechanical pain evoked by movement of inflamed knee joint. Pain 123 (1-2), 193–203. 10.1016/j.pain.2006.02.032 16616417

[B131] Shen C. Pan Z. Wu S. Zheng M. Zhong C. Xin X. (2021). Emodin palliates high-fat diet-induced nonalcoholic fatty liver disease in mice via activating the farnesoid X receptor pathway. J. Ethnopharmacol. 279, 114340. 10.1016/j.jep.2021.114340 34171397

[B132] Shi G. H. Zhou L. (2018). Emodin suppresses angiogenesis and metastasis in anaplastic thyroid cancer by affecting TRAF6-mediated pathways *in vivo* and *in vitro* . Mol. Med. Rep. 18 (6), 5191–5197. 10.3892/mmr.2018.9510 30272291

[B133] Shi Y. Li J. Ren Y. Wang H. Cong Z. Wu G. (2015). Pharmacokinetics and tissue distribution of emodin loaded nanoemulsion in rats. J. drug Deliv. Sci. Technol. 30, 242–249. 10.1016/j.jddst.2015.10.019

[B134] Shia C. S. Hou Y. C. Tsai S. Y. Huieh P. H. Leu Y. L. Chao P. D. (2010). Differences in pharmacokinetics and *ex vivo* antioxidant activity following intravenous and oral administrations of emodin to rats. J. Pharm. Sci. 99 (4), 2185–2195. 10.1002/jps.21978 19921750

[B135] Skopková M. Penesová A. Sell H. Rádiková Z. Vlcek M. Imrich R. (2007). Protein array reveals differentially expressed proteins in subcutaneous adipose tissue in obesity. Obes. (Silver Spring) 15 (10), 2396–2406. 10.1038/oby.2007.285 17925465

[B136] Solis-Herrera C. Triplitt C. Cersosimo E. DeFronzo R. A. (2000). in Pathogenesis of type 2 diabetes mellitus. Editors Feingold K. R. Anawalt B. Blackman M. R. Boyce A. Chrousos G. Corpas E. (South Dartmouth (MA): MDText.com, Inc). Copyright © 2000–2023.

[B137] Song P. Kim J. H. Ghim J. Yoon J. H. Lee A. Kwon Y. (2013). Emodin regulates glucose utilization by activating AMP-activated protein kinase. J. Biol. Chem. 288 (8), 5732–5742. 10.1074/jbc.M112.441477 23303186 PMC3581390

[B138] Song T. Yang Y. Zhou Y. Wei H. Peng J. (2017). GPR120: a critical role in adipogenesis, inflammation, and energy metabolism in adipose tissue. Cell. Mol. Life Sci. 74 (15), 2723–2733. 10.1007/s00018-017-2492-2 28285320 PMC11107682

[B139] Sougiannis A. T. Enos R. T. VanderVeen B. N. Velazquez K. T. Kelly B. McDonald S. (2021). Safety of natural anthraquinone emodin: an assessment in mice. BMC Pharmacol. Toxicol. 22 (1), 9. 10.1186/s40360-021-00474-1 33509280 PMC7845031

[B140] Steppan C. M. Bailey S. T. Bhat S. Brown E. J. Banerjee R. R. Wright C. M. (2001). The hormone resistin links obesity to diabetes. Nature 409 (6818), 307–312. 10.1038/35053000 11201732

[B141] Su X. Peng D. (2020). The exchangeable apolipoproteins in lipid metabolism and obesity. Clin. Chim. Acta 503, 128–135. 10.1016/j.cca.2020.01.015 31981585

[B142] Suffee N. Hlawaty H. Meddahi-Pelle A. Maillard L. Louedec L. Haddad O. (2012). RANTES/CCL5-induced pro-angiogenic effects depend on CCR1, CCR5 and glycosaminoglycans. Angiogenesis 15 (4), 727–744. 10.1007/s10456-012-9285-x 22752444

[B143] Tarasenko T. N. McGuire P. J. (2017). The liver is a metabolic and immunologic organ: a reconsideration of metabolic decompensation due to infection in inborn errors of metabolism (IEM). Mol. Genet. Metab. 121 (4), 283–288. 10.1016/j.ymgme.2017.06.010 28666653 PMC5553615

[B144] Thyagarajan B. Foster M. T. (2017). Beiging of white adipose tissue as a therapeutic strategy for weight loss in humans. Horm. Mol. Biol. Clin. Investig. 31 (2). 10.1515/hmbci-2017-0016 28672737

[B145] Tian N. Gao Y. Wang X. Wu X. Zou D. Zhu Z. (2018). Emodin mitigates podocytes apoptosis induced by endoplasmic reticulum stress through the inhibition of the PERK pathway in diabetic nephropathy. Drug Des. Devel Ther. 12, 2195–2211. 10.2147/dddt.S167405 PMC6047613 30034224

[B146] Tian E. Wang F. Zhao L. Sun Y. Yang J. (2023). The pathogenic role of intestinal flora metabolites in diabetic nephropathy. Front. Physiol. 14, 1231621. 10.3389/fphys.2023.1231621 37469558 PMC10352811

[B147] Tomlinson D. R. (1999). Mitogen-activated protein kinases as glucose transducers for diabetic complications. Diabetologia 42 (11), 1271–1281. 10.1007/s001250051439 10550410

[B148] Tong H. Huang Z. Chen H. Zhou B. Liao Y. Wang Z. (2020). Emodin reverses gemcitabine resistance of pancreatic cancer cell lines through inhibition of IKKβ/NF-κB signaling pathway. Onco Targets Ther. 13, 9839–9848. 10.2147/ott.S253691 33061461 PMC7537840

[B149] Tsai T. Chen C. (1992). Ultraviolet spectrum identification of emodin in rabbit plasma by HPLC and its pharmacokinetics application. Asia Pac. J. Pharmacol. 7 (1), 53–56. https://scholar.nycu.edu.tw/en/publications/ultraviolet-spectrum-identification-of-emodin-in-rabbit-plasma-by .

[B150] Tu C. Gao D. Li X. F. Li C. Y. Li R. S. Zhao Y. L. (2015). Inflammatory stress potentiates emodin-induced liver injury in rats. Front. Pharmacol. 6, 233. 10.3389/fphar.2015.00233 26557087 PMC4615941

[B151] Tzeng T. F. Lu H. J. Liou S. S. Chang C. J. Liu I. M. (2012a). Emodin protects against high-fat diet-induced obesity via regulation of AMP-activated protein kinase pathways in white adipose tissue. Planta Med. 78 (10), 943–950. 10.1055/s-0031-1298626 22673833

[B152] Tzeng T. F. Lu H. J. Liou S. S. Chang C. J. Liu I. M. (2012b). Emodin, a naturally occurring anthraquinone derivative, ameliorates dyslipidemia by activating AMP-activated protein kinase in high-fat-diet-fed rats. Evid. Based Complement. Altern. Med. 2012, 781812. 10.1155/2012/781812 PMC3357974 22649478

[B153] Unger R. H. Orci L. (2001). Diseases of liporegulation: new perspective on obesity and related disorders. Faseb J. 15 (2), 312–321. 10.1096/fj.00-0590 11156947

[B154] Wahi D. Soni D. Grover A. (2021). A double-edged sword: the anti-cancer effects of emodin by inhibiting the redox-protective protein MTH1 and augmenting ROS in NSCLC. J. Cancer 12 (3), 652–681. 10.7150/jca.41160 33403025 PMC7778552

[B155] Wang J. Huang H. Liu P. Tang F. Qin J. Huang W. (2006). Inhibition of phosphorylation of p38 MAPK involved in the protection of nephropathy by emodin in diabetic rats. Eur. J. Pharmacol. 553 (1-3), 297–303. 10.1016/j.ejphar.2006.08.087 17074319

[B156] Wang Y. J. Huang S. L. Feng Y. Ning M. M. Leng Y. (2012). Emodin, an 11β-hydroxysteroid dehydrogenase type 1 inhibitor, regulates adipocyte function *in vitro* and exerts anti-diabetic effect in ob/ob mice. Acta Pharmacol. Sin. 33 (9), 1195–1203. 10.1038/aps.2012.87 22922341 PMC4003114

[B157] Wang S. Li X. Guo H. Yuan Z. Jiang Z. (2016). Emodin alleviates hepatic steatosis by inhibiting SREBP1 activity via the CaMKK-AMPK-mTOR-p70S6K signaling pathway: emodin alleviates NAFLD by CaMKK-AMPK-mTOR-SREBP1. Hepatology Res. 47 (7). 10.1111/hepr.12788 27492505

[B158] Wang S. Li X. Guo H. Yuan Z. Wang T. Zhang L. (2017a). Emodin alleviates hepatic steatosis by inhibiting sterol regulatory element binding protein 1 activity by way of the calcium/calmodulin-dependent kinase kinase-AMP-activated protein kinase-mechanistic target of rapamycin-p70 ribosomal S6 kinase signaling pathway. Hepatol. Res. 47 (7), 683–701. 10.1111/hepr.12788 27492505

[B159] Wang X. Lin B. Nie L. Li P. (2017b). microRNA-20b contributes to high glucose-induced podocyte apoptosis by targeting SIRT7. Mol. Med. Rep. 16 (4), 5667–5674. 10.3892/mmr.2017.7224 28849008

[B160] Wang K. J. Meng X. Y. Chen J. F. Wang K. Y. Zhou C. Yu R. (2021). Emodin induced necroptosis and inhibited glycolysis in the renal cancer cells by enhancing ROS. Oxid. Med. Cell. Longev. 2021, 8840590. 10.1155/2021/8840590 33532038 PMC7837784

[B161] Wang M. Zhang Z. Ruan P. Zhang G. Xiao C. Wang Y. (2022). Emodin-induced hepatotoxicity is enhanced by 3-methylcholanthrene through activating aryl hydrocarbon receptor and inducing CYP1A1 *in vitro* and *in vivo* . Chem. Biol. Interact. 365, 110089. 10.1016/j.cbi.2022.110089 35934134

[B162] Wang Y. Dong N. Zhou Y. Li H. Qin G. Li H. (2023). Effects of emodin on protein expression related to autophagy of interstitial cells of cajal in diabetic rats. Chem. Pharm. Bull. (Tokyo) 71 (2), 129–133. 10.1248/cpb.c22-00596 36464270

[B163] Wei W. Wang J. Hu Y. Chen S. Liu J. (2023). Emodin reverses resistance to gemcitabine in pancreatic cancer by suppressing stemness through regulation of the epithelial-mesenchymal transition. Exp. Ther. Med. 25 (1), 7. 10.3892/etm.2022.11706 36545274 PMC9748633

[B164] Wen J. Bao Z. Li L. Liu Y. Wei B. Ye X. (2023). Qiangguyin inhibited fat accumulation in OVX mice through the p38 MAPK signaling pathway to achieve anti-osteoporosis effects. Biomed. Pharmacother. 158, 114122. 10.1016/j.biopha.2022.114122 36566522

[B165] Wu X. Xiao B. (2023). TDAG51 attenuates impaired lipid metabolism and insulin resistance in gestational diabetes mellitus through SREBP-1/ANGPTL8 pathway. Balk. Med. J. 40 (3), 175–181. 10.4274/balkanmedj.galenos.2023.2022-8-61 PMC10175879 36960944

[B166] Wu Z. Chen Q. Ke D. Li G. Deng W. (2014). Emodin protects against diabetic cardiomyopathy by regulating the AKT/GSK-3β signaling pathway in the rat model. Molecules 19 (9), 14782–14793. 10.3390/molecules190914782 25232702 PMC6271268

[B167] Wu L. Han W. Chen Y. Zhang T. Liu J. Zhong S. (2018). Gender differences in the hepatotoxicity and toxicokinetics of emodin: the potential mechanisms mediated by UGT2B7 and MRP2. Mol. Pharm. 15 (9), 3931–3945. 10.1021/acs.molpharmaceut.8b00387 30011215

[B168] Wu W. Lu P. Huang Y. Zhu Z. Li C. Liu Y. (2022). Emodin regulates the autophagy via the miR-371a-5p/PTEN axis to inhibit hepatic malignancy. Biochem. Biophys. Res. Commun. 619, 1–8. 10.1016/j.bbrc.2022.06.006 35724456

[B169] Xiao D. Hu Y. Fu Y. Wang R. Zhang H. Li M. (2019). Emodin improves glucose metabolism by targeting microRNA-20b in insulin-resistant skeletal muscle. Phytomedicine 59, 152758. 10.1016/j.phymed.2018.11.018 31004884

[B170] Xie R. Liu M. Li S. (2019). Emodin weakens liver inflammatory injury triggered by lipopolysaccharide through elevating microRNA-145 *in vitro* and *in vivo* . Artif. Cells Nanomed Biotechnol. 47 (1), 1877–1887. 10.1080/21691401.2019.1614015 31079494

[B171] Xu H. Li J. Song S. Xiao Z. Chen X. Huang B. (2021a). Effective inhibition of coronavirus replication by Polygonum cuspidatum. Front. Biosci. (Landmark Ed. 26 (10), 789–798. 10.52586/4988 34719206

[B172] Xu Q. Wang M. Guo H. Liu H. Zhang G. Xu C. (2021b). Emodin alleviates severe acute pancreatitis-associated acute lung injury by inhibiting the cold-inducible RNA-binding protein (CIRP)-Mediated activation of the NLRP3/IL-1β/CXCL1 signaling. Front. Pharmacol. 12, 655372. 10.3389/fphar.2021.655372 33967799 PMC8103163

[B173] Xue J. Ding W. Liu Y. (2010). Anti-diabetic effects of emodin involved in the activation of PPARgamma on high-fat diet-fed and low dose of streptozotocin-induced diabetic mice. Fitoterapia 81 (3), 173–177. 10.1016/j.fitote.2009.08.020 19699280

[B174] Yan X. Gu S. Shi Y. Cui X. Wen S. Ge J. (2017). The effect of emodin on *Staphylococcus aureus* strains in planktonic form and biofilm formation *in vitro* . Arch. Microbiol. 199 (9), 1267–1275. 10.1007/s00203-017-1396-8 28616631

[B175] Yang Y. Shang W. Zhou L. Jiang B. Jin H. Chen M. (2007). Emodin with PPARγ ligand-binding activity promotes adipocyte differentiation and increases glucose uptake in 3T3-Ll cells. Biochem. Biophysical Res. Commun. 353 (2), 225–230. 10.1016/j.bbrc.2006.11.134 17174269

[B176] Yang J. Zeng Z. Wu T. Yang Z. Liu B. Lan T. (2013). Emodin attenuates high glucose-induced TGF-β1 and fibronectin expression in mesangial cells through inhibition of NF-κB pathway. Exp. Cell. Res. 319 (20), 3182–3189. 10.1016/j.yexcr.2013.10.006 24140264

[B177] Yang F. Yuan P. W. Hao Y. Q. Lu Z. M. (2014). Emodin enhances osteogenesis and inhibits adipogenesis. BMC Complement. Altern. Med. 14, 74. 10.1186/1472-6882-14-74 24565373 PMC3974048

[B178] Yang X. Zhang Y. Liu Y. Chen C. Xu W. Xiao H. (2018). Emodin induces liver injury by inhibiting the key enzymes of FADH/NADPH transport in rat liver. Toxicol. Res. (Camb) 7 (5), 888–896. 10.1039/c7tx00307b 30310665 PMC6116728

[B179] Yang Y. Jiang Z. Zhuge D. (2019). Emodin attenuates lipopolysaccharide-induced injury via down-regulation of miR-223 in H9c2 cells. Int. Heart J. 60 (2), 436–443. 10.1536/ihj.18-048 30745529

[B180] Yang H. Y. Wu J. Lu H. Cheng M. L. Wang B. H. Zhu H. L. (2023). Emodin suppresses oxaliplatin-induced neuropathic pain by inhibiting COX2/NF-κB mediated spinal inflammation. J. Biochem. Mol. Toxicol. 37 (1), e23229. 10.1002/jbt.23229 36184831

[B181] Yong W. Hongbin L. Jing W. Jing Z. Ning T. Lijie B. (2020). Associations of changes in serum inflammatory factors, MMP-3, 25 (OH)D and intestinal flora with osteoporosis and disease activity in rheumatoid arthritis patients. Clin. Lab. 66 (12). 10.7754/Clin.Lab.2020.200242 33337838

[B182] Yu B. Pu Y. Liu J. Liao J. Chen K. Zhang J. (2020a). Targeted delivery of emodin to adipocytes by aptamer-functionalized PEG-PLGA nanoparticles *in vitro* . J. Drug Deliv. Sci. Technol. 57, 101739. 10.1016/j.jddst.2020.101739

[B183] Yu L. Gong L. Wang C. Hu N. Tang Y. Zheng L. (2020b). Radix polygoni multiflori and its main component emodin attenuate non-alcoholic fatty liver disease in zebrafish by regulation of AMPK signaling pathway. Drug Des. Devel Ther. 14, 1493–1506. 10.2147/dddt.S243893 PMC7167271 32346285

[B184] Yu F. Yu N. Peng J. Zhao Y. Zhang L. Wang X. (2021). Emodin inhibits lipid accumulation and inflammation in adipose tissue of high-fat diet-fed mice by inducing M2 polarization of adipose tissue macrophages. Faseb J. 35 (7), e21730. 10.1096/fj.202100157RR 34110631

[B185] Zhang S. L. Filep J. G. Hohman T. C. Tang S. S. Ingelfinger J. R. Chan J. S. (1999). Molecular mechanisms of glucose action on angiotensinogen gene expression in rat proximal tubular cells. Kidney Int. 55 (2), 454–464. 10.1046/j.1523-1755.1999.00271.x 9987070

[B186] Zhang Y. Lee F. Y. Barrera G. Lee H. Vales C. Gonzalez F. J. (2006). Activation of the nuclear receptor FXR improves hyperglycemia and hyperlipidemia in diabetic mice. Proc. Natl. Acad. Sci. U. S. A. 103 (4), 1006–1011. 10.1073/pnas.0506982103 16410358 PMC1347977

[B187] Zhang Y. Fan S. Hu N. Gu M. Chu C. Li Y. (2012). Rhein reduces fat weight in db/db mouse and prevents diet-induced obesity in C57Bl/6 mouse through the inhibition of PPARγ signaling. PPAR Res. 2012, 374936. 10.1155/2012/374936 23049539 PMC3463192

[B188] Zhang Y. Xu Y. Qi Y. Xu L. Song S. Yin L. (2017). Protective effects of dioscin against doxorubicin-induced nephrotoxicity via adjusting FXR-mediated oxidative stress and inflammation. Toxicology 378, 53–64. 10.1016/j.tox.2017.01.007 28082111

[B189] Zhang T. He X. Sun L. Wang D. Zhang S. Mao J. (2021). Insight into the practical models for prediciting the essential role of the cytochrome P450-mediated biotransformation in emodin-associated hepatotoxicity. Toxicology 462, 152930. 10.1016/j.tox.2021.152930 34492313

[B190] Zhang F. Y. Li R. Z. Xu C. Fan X. X. Li J. X. Meng W. Y. (2022a). Emodin induces apoptosis and suppresses non-small-cell lung cancer growth via downregulation of sPLA2-IIa. Phytomedicine 95, 153786. 10.1016/j.phymed.2021.153786 34785104

[B191] Zhang M. Lian B. Zhang R. Guo Y. Zhao J. He S. (2022b). Emodin ameliorates intestinal dysfunction by maintaining intestinal barrier integrity and modulating the microbiota in septic mice. Mediat. Inflamm. 2022, 5026103. 10.1155/2022/5026103 PMC9168211 35677734

[B192] Zhang X. Qin Q. Lv X. Wang Y. Luo F. Xue L. (2022c). Natural emodin reduces myocardial ischemia/reperfusion injury by modulating the RUNX1/miR-142-3p/DRD2 pathway and attenuating inflammation. Exp. Ther. Med. 24 (6), 745. 10.3892/etm.2022.11681 36561980 PMC9748643

[B193] Zhao X. Y. Qiao G. F. Li B. X. Chai L. M. Li Z. Lu Y. J. (2009). Hypoglycaemic and hypolipidaemic effects of emodin and its effect on L-type calcium channels in dyslipidaemic-diabetic rats. Clin. Exp. Pharmacol. Physiol. 36 (1), 29–34. 10.1111/j.1440-1681.2008.05051.x 18785977

[B194] Zhao Q. H. Wang S. G. Liu S. X. Li J. P. Zhang Y. X. Sun Z. Y. (2013). PPARγ forms a bridge between DNA methylation and histone acetylation at the C/EBPα gene promoter to regulate the balance between osteogenesis and adipogenesis of bone marrow stromal cells. Febs J. 280 (22), 5801–5814. 10.1111/febs.12500 23981481

[B195] Zheng X. Y. Yang S. M. Zhang R. Wang S. M. Li G. B. Zhou S. W. (2019). Emodin-induced autophagy against cell apoptosis through the PI3K/AKT/mTOR pathway in human hepatocytes. Drug Des. Devel Ther. 13, 3171–3180. 10.2147/dddt.S204958 PMC6734549 31564833

[B196] Zheng K. Lv B. Wu L. Wang C. Xu H. Li X. (2022). Protecting effect of emodin in experimental autoimmune encephalomyelitis mice by inhibiting microglia activation and inflammation via Myd88/PI3K/Akt/NF-κB signalling pathway. Bioengineered 13 (4), 9322–9344. 10.1080/21655979.2022.2052671 35287559 PMC9161934

[B197] Zhou Q. Xiang H. Liu H. Qi B. Shi X. Guo W. (2021). Emodin alleviates intestinal barrier dysfunction by inhibiting apoptosis and regulating the immune response in severe acute pancreatitis. Pancreas 50 (8), 1202–1211. 10.1097/mpa.0000000000001894 34714285 PMC8565508

[B198] Zhou J. Zhou D. Xie H. Zhou Y. Chen W. Lian D. (2023a). Protective effect of emodin on Streptococcus pneumoniae and matrix-assisted laser desorption time-of-flight mass spectrometry for serotype detection. Mater. Express 13 (5), 896–903. 10.1166/mex.2023.2419

[B199] Zhou L. Hu X. Han C. Niu X. Han L. Yu H. (2023b). Comprehensive investigation on the metabolism of emodin both *in vivo* and *in vitro* . J. Pharm. Biomed. Anal. 223, 115122. 10.1016/j.jpba.2022.115122 36327583

[B200] Zhou X. Yeasmin Khusbu F. Xie Y. Yang P. (2023c). Emodin-induced necroptosis in prostate cancer cells via the mitochondrial fission HSP90/MLKL/PGAM pathway. Chem. Biodivers. 20 (6), e202201130. 10.1002/cbdv.202201130 37062702

[B201] Zhu S. Wang Y. Wang X. Li J. Hu F. (2014). Emodin inhibits ATP-induced IL-1β secretion, ROS production and phagocytosis attenuation in rat peritoneal macrophages via antagonizing P2X₇ receptor. Pharm. Biol. 52 (1), 51–57. 10.3109/13880209.2013.810648 24028150

[B202] Zhu X. Guo S. Zhang M. Bai X. (2023). Emodin protects against apoptosis and inflammation by regulating reactive oxygen species-mediated NF-κB signaling in interleukin-1β-stimulated human nucleus pulposus cells. Hum. Exp. Toxicol. 42, 9603271221138552. 10.1177/09603271221138552 36598795

